# Enhanced Strontium Removal through Microbially Induced Carbonate Precipitation by Indigenous Ureolytic Bacteria

**DOI:** 10.1021/acsearthspacechem.3c00252

**Published:** 2024-02-26

**Authors:** Matthew White-Pettigrew, Samuel Shaw, Lewis Hughes, Christopher Boothman, James Graham, Liam Abrahamsen-Mills, Katherine Morris, Jonathan R. Lloyd

**Affiliations:** ∥ Research Centre for Radwaste Disposal and Williamson Research Centre for Molecular Environmental Science, Department of Earth and Environmental Sciences, The University of Manchester, Manchester M13 9PL, United Kingdom; ‡ National Nuclear Laboratory, Warrington, Cheshire WA3 6AE, United Kingdom

**Keywords:** radiostrontium, carbonate, sediments, bioremediation, biomineralisation, groundwater

## Abstract

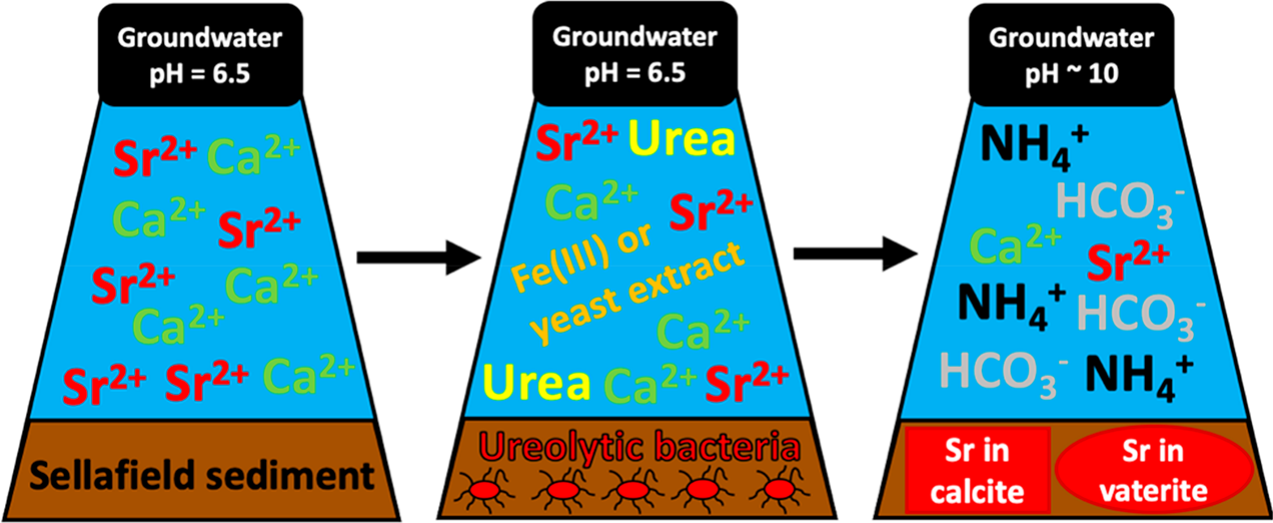

Microbial ureolysis offers the potential to remove metals including Sr^2+^ as carbonate minerals via the generation of alkalinity coupled to NH_4_^+^ and HCO_3_^–^ production. Here, we investigated the potential for bacteria, indigenous to sediments representative of the U.K. Sellafield nuclear site where ^90^Sr is present as a groundwater contaminant, to utilize urea in order to target Sr^2+^-associated (Ca)CO_3_ formation in sediment microcosm studies. Strontium removal was enhanced in most sediments in the presence of urea only, coinciding with a significant pH increase. Adding the biostimulation agents acetate/lactate, Fe(III), and yeast extract to further enhance microbial metabolism, including ureolysis, enhanced ureolysis and increased Sr and Ca removal. Environmental scanning electron microscopy analyses suggested that coprecipitation of Ca and Sr occurred, with evidence of Sr associated with calcium carbonate polymorphs. Sr *K*-edge X-ray absorption spectroscopy analysis was conducted on authentic Sellafield sediments stimulated with Fe(III) and quarry outcrop sediments amended with yeast extract. Spectra from the treated Sellafield and quarry sediments showed Sr^2+^ local coordination environments indicative of incorporation into calcite and vaterite crystal structures, respectively. 16S rRNA gene analysis identified ureolytic bacteria of the genus *Sporosarcina* in these incubations, suggesting they have a key role in enhancing strontium removal. The onset of ureolysis also appeared to enhance the microbial reduction of Fe(III), potentially via a tight coupling between Fe(III) and NH_4_^+^ as an electron donor for metal reduction. This suggests ureolysis may support the immobilization of ^90^Sr via coprecipitation with insoluble calcium carbonate and cofacilitate reductive precipitation of certain redox active radionuclides, e.g., uranium.

## Introduction

Strontium-90 (half-life = 28.8 years) comprises a significant portion of the radioactivity associated with fission products within a variety of radioactive wastes.^1−6^ In addition, ^90^Sr is present as a subsurface environmental contaminant at a number of nuclear sites across the world, including Hanford, USA,^7,8^ and Mayak, Russia.^9,10^ For example, at Sellafield, U.K., the unintentional release of ^90^Sr has generated radioactive groundwater plumes which originate from storage units, as well as nitrate-neutralized wastes.^11^ In addition, the environmental contamination by ^90^Sr as a consequence of the accidents at Chernobyl, Ukraine, and Fukushima Daichii Nuclear Power Plant, Japan, are well documented to have polluted water bodies and soils at these sites.^12−18^ In order to remediate ^90^Sr subsurface contamination, it is necessary to develop a toolkit of effective and sustainable remediation technologies in order to limit its migration and minimize potential environmental harm.

In the hydrosphere, ^90^Sr persists as Sr^2+^ and exhibits similar biogeochemical behavior to Ca^2+^ in the aqueous phase, as they both possess divalent charge and have similar ionic radii.^19,20^ At circumneutral pH, the mobility of Sr^2+^ in soil and groundwater systems is typically controlled by outer-sphere adsorption and ion exchange to phyllosilicate clays and iron oxide mineral particles,^21−23^ with the degree of adsorption controlled by a number of factors including pH and ionic strength.^24,25^ The pH point of zero charge (pH_pzc_) of these naturally occurring minerals ranges from approximately 2 to 5 for clay minerals (e.g., kaolinite and Illite)^26−28^ to approximately 5 to 7 for iron oxides, e.g., goethite (α-FeOOH) and lepidocrocite (γ-FeOOH).^29,30^ At pH > pH_pzc_, the negatively charged surface sites enhance the adsorption of cations such as Sr^2+^. At the same time, increasing ionic strength often leads to a reduction in Sr^2+^ uptake due to the competition for surface sites from other cations.^31−11^ This can be particularly challenging given radioactive plumes in the subsurface often contain elevated ionic strength attributed to released liquors.^34^ At Sellafield, U.K., the transport of ^90^Sr is hypothesized to be mediated largely by an adsorption process given that the groundwater pH is between 6 and 8.^35^ Modeling the partition coefficient (*K*_d_, a measure of contaminant mobility) for ^90^Sr sorption to Sellafield sediments has indicated its uptake is significantly reduced in high ionic strength MAGNOX tank liquors of pH 9–11.5 (*K*_d_ ∼ 40 L/kg) compared with circumneutral groundwaters at pH 6–8 (*K*_d_ ∼ 10^3^ L/kg).^11^

The migration of Sr^2+^ in groundwaters can be limited via incorporation into mineral phases that actively form in the subsurface, via either biotic or abiotic processes. The incorporation of Sr^2+^ into Ca^2+^-bearing (bio)minerals results in a far more recalcitrant, and less labile, end point compared with Sr^2+^ outer-sphere adsorbed to minerals surfaces. However, few studies have investigated Sr^2+^ sequestration into Ca^2+^ (bio)minerals forming under conditions applicable to contaminated land environments. The partitioning of Sr^2+^ into calcium carbonate minerals occurs within all environmentally relevant polymorphs including calcite,^37^ aragonite,^38^ and vaterite.^39,40^ Studies investigating strontium uptake within chemically precipitated calcite have reported concentrations ranging from several hundred ppm^41,42^ to several thousand ppm.^43^ While biogenic aragonite^44,45^ and calcite^46,47^ have been shown to contain up to several thousand ppm of Sr, aragonite is able to retain far higher Sr concentrations.^48^ Mechanistically, this is because the 9-fold coordination of divalent cations in aragonite allows for easier accommodation of larger cations, such as Sr^2+^, than the 6-fold coordination environment in calcite.^49^ Furthermore, aragonite has a lower crystal symmetry compared to calcite, which may contribute to increasing the availability of lattice sites for Sr incorporation.^50,51^ Sr *K*-edge X-ray absorption near edge spectroscopy (XANES) and extended X-ray absorption fine structure (EXAFS) are key methods for understanding the Sr atomic scale local environment and the mechanism of uptake into calcium carbonate minerals. The use of EXAFS analysis to identify Sr–Ca coordination at radial distances approximately 3.9–4.1 Å in calcite^52,53^ and aragonite^54,55^ and 4.2 Å in vaterite^43,53^ indicates the substitution of Sr^2+^ for Ca^2+^ into the crystal structure of calcium carbonate as the uptake mechanism.

Microbially induced calcite precipitation (MICP) leads to the incorporation of Sr into calcium carbonate, which occurs in a variety of natural settings including marine systems and terrestrial soils.^56^ The MICP process is initiated by the metabolic generation of sufficient elevated alkalinity and high pH conditions required to precipitate calcium carbonate. Ureolysis is capable of instigating MICP, which involves the degradation of urea [CO(NH_2_)_2_] by bacterial urease enzymes to form ammonia (NH_3_) and carbonic acid (H_2_CO_3_). Under circumneutral groundwater conditions, NH_3_ and H_2_CO_3_ then equilibrate as ammonium (NH_4_^+^) and bicarbonate (HCO_3_^–^), respectively [eqs 1–3^57^].
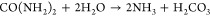
1
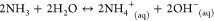
2
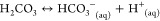
3

NH_4_^+^ formation increases the pH of a solution, and the breakdown of urea to bicarbonate increases the alkalinity. Under geochemical conditions commonly found in natural aquatic environments where Ca^2+^ is present, production of HCO_3_^–^ induces conditions conducive to calcium carbonate precipitation [eq 4^58^].
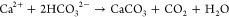
4

Industrial applications for MICP via urea-hydrolyzing (ureolytic) microbial communities indigenous to soils and groundwater include: (i) the sealing of rock permeability and fracture networks in order to retard the migration of aqueous contaminants,^59^ (ii) the repair and reinforcement of rocks and cement,^60,61^ (iii) the mitigation of arid soil erosion.^62^ Despite the identification of indigenous ureolytic species in various (contaminated) natural environments,^63−65^ few studies have successfully demonstrated ureolytic strontium removal through calcite precipitation under environmentally relevant conditions. Past work including a study of Sr removal by MICP used a carbon-rich medium to enhance the activity of ureolytic pure cultures during incubation with aqueous Sr^2+^.^58^ The study showed that 80% of the initial aqueous Sr was associated with calcium carbonate only 4 h after the onset of ureolysis, with near-total Sr uptake achieved after 24 h. Other studies have focused on pure cultures of *Bacillus pasteurii* to remediate ^90^Sr in simulated groundwaters analogous to those within the Snake River Plain aquifer, USA,^66,67^ a radionuclide-impacted water body underlying a licensed nuclear site. Both studies showed the immediate coremoval of Sr and Ca at the start of ureolysis, which was later attributed to calcite precipitation by a combination of scanning electron microscopy (SEM) and X-ray absorption spectroscopy (XAS). A follow-on study by Fujita et al.^65^ evaluated the potential for indigenous urease activity within sediments obtained from the ^90^Sr-contaminated subsurface at Hanford to remediate aqueous ^90^Sr contamination. Quantifying *ureC* copies (mL^–1^), ureolysis rates (nmol L^–1^ h^–1^), and ureolytic MPN (cells mL^–1^) enabled a site-specific geochemical model to be generated for strontium remediation via urease-driven calcite precipitation. The distribution of ureolytic activity between the sediments obtained at different depths was heterogeneous. In spite of the variability, model results indicated the microbial community at the Hanford subsurface is capable of MICP via ureolysis and concurrently remediating ^90^Sr contamination via calcite coprecipitation. The model also determined that the onset of ureolysis immediately precipitated Sr-containing calcite and that it was possible to sequester virtually all of the 0.25 ppm of Sr^2+^ from groundwater after 150 days of ureolysis. To date, this is the only study that we are aware of that has modeled MICP in sediment systems from radionuclide-impacted land for urease-facilitated strontium remediation. However, no experiments evidencing enhanced Sr^2^ removal were performed. Further studies of ureolysis using real sediments and microbial communities under a range of geochemical conditions (e.g., aerobic and anaerobic) are required in order to further evaluate the applicability of this remediation strategy to a range of contaminated sites.

This current study aims to demonstrate enhanced Sr^2+^ removal through MICP generated by indigenous ureolytic bacteria in sediments representative of the subsurface at the Sellafield, UK nuclear licensed site. The potential for native microbial communities to hydrolyze urea with associated Sr removal was investigated using a series of sediment microcosms. In addition, the incubations were amended with various biostimulants, e.g., acetate, lactate, Fe(III), and yeast extract under either aerobic or anaerobic conditions. The reaction end-products were characterized by ESEM, XAS, and energy-dispersive X-ray spectroscopy (EDS) to determine nature and speciation and fate of Sr in the remediated solid phase. Overall, the project assessed the viability of urea addition to groundwater/sediments contaminated with Sr^2+^ in the context of future *in situ* MICP remediation strategies. The results indicated that the native microbial communities were capable of degrading urea, concomitantly increasing the pH of Sellafield-representative groundwater and facilitating increased levels of Sr removal into biogenic calcium carbonate.

## Materials and Methods

### Sediment Collection

Four different sediment lithologies characterized by quaternary glaciogenic formations were used in the sediment microcosms. The first were sand-rich sediments obtained from Peel Place Quarry (PPQ), Cumbia, UK that represent the Peel Place Sand and Gravel Member, a lithology identified in the Sellafield subsurface.^68,69^ The second were more clay-rich tills obtained from the bed of the Calder River (CR) approximately 2 km north of the Sellafield Site, a lithology known to surround fluvial systems in the Sellafield district.^70^ After collection, these two sediments were immediately transferred to sealed, sterile HDPE Ziplock bags to the University of Manchester and kept in the dark at 10 °C prior to experimentation. Bulk mineralogy of these sediments was determined by X-ray diffraction (XRD, Bruker D8 Advance). The final two samples are aged sediments named as RB23 and RB27, extracted during the 2009/2010 and 2012 Sellafield site drilling programs, respectively, and investigated in 2014 for their ability to support microbial reduction of Fe(III) to Fe(II) and U(VI) to U(IV) simultaneously in ref (71). Samples RB23 and RB27 were transported to the University of Manchester in 2011 and 2012, respectively, and stored under refrigeration at 10 °C in the dark until use in this study. XRD analysis showed that the sediments were all predominantly composed of quartz but also contained phyllosilicate clays (e.g., clinochlore and muscovite) and feldspars (e.g., albite, orthoclase, and microcline) (Figure S1).^71^

### Sr Bioremediation with Urea

Sediment microcosm experiments were incubated for 25 days in both open (air equilibrated) and closed systems to investigate whether microbial communities indigenous to various sediments, representative of the Sellafield subsurface, were capable of enhancing strontium removal via MICP, initiated by the breakdown of urea under open and closed conditions (Table 1). Sediment microcosms were constructed with a 1:10 ratio of sediment to synthetic groundwater–water representative of the Sellafield region.^140^ Prior to sediment microcosm incubations, 100 ppm of Sr was added as strontium dichloride hexahydrate (SrCl_2_·6H_2_O) to artificial groundwater (AGW) of the following composition (g/L): MgSO_4_·7H_2_O, 0.05; CaSO_4_, 0.008; KCl, 0.01; NaCl, 0.012; CaCl_2_·2H_2_O, 0.092; NaNO_3_, 0.028; NaHCO_3_, 0.08. After 7 days, the microcosms were spiked with 88 ppm of Sr^2+^ and 80 ppm of Ca^2+^ to instigate further Sr^2+^ coprecipitation with calcium carbonate in the urea-bearing systems and display differences in Sr/Ca removal rates in the urea-free controls and urea-amended systems more clearly. In urea-amended experiments, it was added in excess (∼400 mM) so as to clearly evidence the ureolytic capabilities of the native bacterial communities. The microcosms containing 5 mM acetate/lactate + 10 mM amorphous Fe(III) gel were to investigate whether any potential iron reduction over this time period impacted strontium removal.^72,73^ Additional microcosms were constructed using Sellafield sediments RB23 and RB27 to assess whether sole urea amendments enhanced the microbial reduction of Fe(III) to Fe(II).

**Table 1:** Outline of the Treatment Systems Investigating Sr Bioremediation in This Study

system notation	treatment description	open or closed	rationale
A	Urea-free control	Open	Sr removal in this system will be compared to experimental urea-bearing systems.
B	Urea only	Open	When considering perspective *in situ* bioremediation strategies for contaminated land, minimizing the number of compounds in amendment solutions may be ideal.
C	Urea + 5 mM acetate + 5 mM lactate	Closed	Electron donors widely oxidized by anaerobic soil bacteria, coupled to reduction of redox active species. Such biostimulation may help establish an anaerobic ureolytic community within Fe(III)-bearing sediments.
D	Urea + 1 g/L yeast extract	Open	Ureolytic microbial communities indigenous to soils have been successfully stimulated using 1 g L^–1^ yeast extract, which provides trace nutrients.^62^
E	Urea + 10 mM amorphous Fe(III) as ferrihydrite	Closed	Fe(III) addition sought to stimulate the microbial Fe(III) reduction in the presence of oxidizable residual organic carbon in sediments. As with acetate and lactate, adding a bioavailable and redox-active compound aimed to promote anaerobic metabolic activity

### Geochemical Measurements

Aliquots from each sediment microcosm were periodically extracted in order monitor the biogeochemical conditions at selected time points. Sediment slurries were digested in 0.5 N HCl ± 6 M hydroxylamine–HCl for the determination of bioavailable Fe(II) and total bioavailable Fe [Fe(T)], respectively, using the ferrozine assay.^74,75^ After centrifugation (16 200*g*, 5 min), supernatant samples were analyzed to measure aqueous Sr^2+^ and Ca^2+^ by ICP-MS (Agilent 7500cx) as well as pH. Supernatant samples were also used for the colorimetric determination of urea,^67,76^ measured at 422 nm using a Jenway 6715 UV–vis spectrophotometer.

### Solid-Phase Characterization

At the final time point investigating strontium removal, homogeneous sediment samples were removed from selected microcosms and analyzed by X-ray absorption spectroscopy (XAS) at the DIAMOND Lightsource, Harwell, UK. Sr *K*-edge (16,105.55 eV) X-ray absorption near edge spectroscopy (XANES) and extended X-ray absorption fine structure (EXAFS) analyses were conducted to determine the coordination environment of Sr^2+^ in the samples studied. The samples were stored at −80 °C prior to analysis on Beamline B18, where they were loaded into a liquid N_2_-cooled cryostat. XANES and EXAFS data were obtained in fluorescence mode using a 36-element solid state Ge detector. Data were background subtracted and drift corrected using the software package ATHENA.^77^ EXAFS modeling was conducted using the package ARTEMIS.^77^ The F-test was used to statistically determine whether the addition of a coordination path improved the fit of a model relative to the spectral data.^78^

Further solid-phase characterization of the Sr-bearing precipitates within selected samples was conducted using environmental scanning electron microscopy (ESEM) and associated elemental mapping. This involved mounting a small quantity of dried sediment slurry onto aluminum stubs using carbon tape. ESEM images with energy dispersive X-ray analysis (EDS) were collected using a FEI Quanta 650 FEG ESEM in low vacuum mode.

### Geochemical Modeling

The impact of ureolysis on the solubility of various carbonate minerals was modeled using the geochemical modeling program PHREEQC.^79^ The applied thermodynamic database was “Thermochimie.dat” (version 10a) as it is most suitable for geochemical calculations involving radionuclides.^80^ The model uses the first-order urea degradation equation , where the urea degradation rate *k*_urea_ = 0.04 day ^–1^ represents the activity of biostimulated ureolytic communities indigenous to natural groundwater.^59^

### Microbial Analyses

DNA extractions were performed on selected sediment slurries at the start of the experiment and 17 days after incubation in order to compare changes in the microbial community with urea and biostimulant addition. Extractions were achieved using a PowerSoil DNA isolation kit (MO Bio, USA). Sequencing of the 16S rRNA genes was conducted using the Illumina MiSeq platform (Illumina, San Diego, CA, USA). Bioinformatics was performed via a bespoke pipeline, outlined in the Supporting Information.^81,82^

## Results and Discussion

### Biogeochemical Changes to Sediment Microcosms

**Figure 1:**
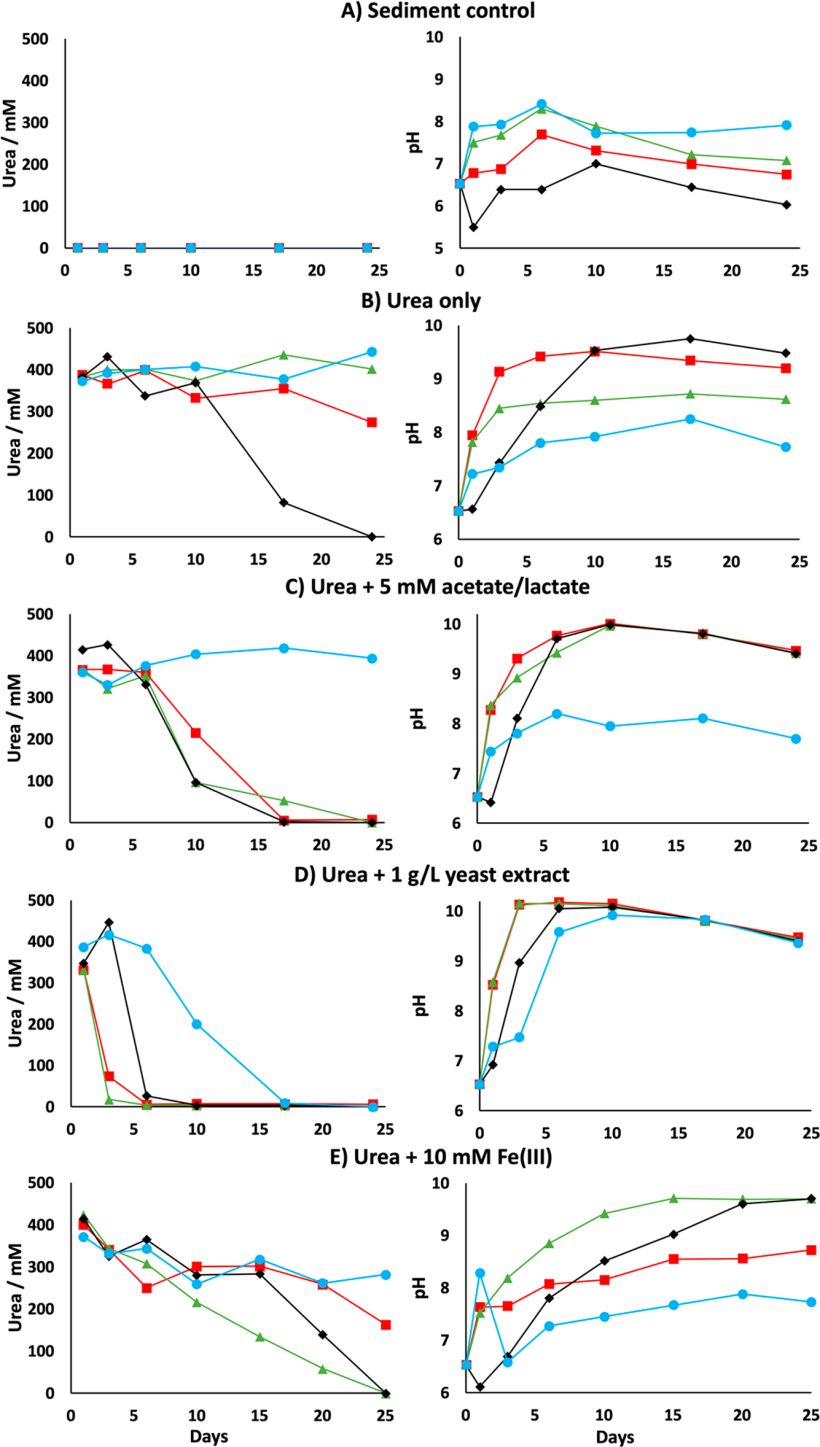
Geochemical changes observed in biostimulated microcosms containing Sellafield-relevant sediments CR (black diamonds), PPQ (blue circles), RB23 (red squares), and RB27 (green triangles).

Within 6 days of incubation, the urea-free controls (System A) for sediments PPQ and RB27 equilibrated at pH 8.3, (Figure 1a), whereas sediments CR and RB23 equilibrated at pH 6.4 and 7.7, respectively. In contrast, by day 6 in urea-only microcosms (System B), RB23, RB27, and CR microcosms showed clearly elevated pH of 9.4, 8.5, and 9.5, respectively (Figure 1b). In these three microcosms, the pH increased from 6.5 (corresponding to that of Sellafield groundwater) to ≥8.5 and then either stabilized or continued to increase thereafter. System B for PPQ sediment was the only incubation that did not display a greater pH increase compared the respective urea-free control. For urea-only incubations, CR and RB23 were the only sediments to generate a pH increase >9 and display clear evidence of urea degradation. RB27 stabilized at pH 8.5 in System B, likely caused by the presence of urea, compared with a decline from pH 8.3 to 7.1 in urea-free controls. CR sediments observed the largest increase in pH for urea-only amendments (pH 9.8), coinciding with complete urea removal by day 24. Urea degradation by ureolysis generates ammonium, responsible for elevating the pH of aqueous systems. The decomposition of urea and concomitant increase in (and stabilization of) pH thus strongly implies that ureolysis was the degradation mechanism for urea. For urea plus acetate/lactate amendments (System C), complete urea degradation coincided with an increase in pH above 9.4 by day 6 in sediments RB23, RB27, and CR. A similar trend was observed with urea and additional yeast extract (System D), where an increase in pH to ≥9.6 was observed in all sediments by day 6. In Systems C and D, carbon amendments appeared to enhance the rates and extents of ureolysis in all sediments compared to urea-only amendments (Figure 1c,d). Notably, the addition of acetate and lactate failed to stimulate urea decomposition in the PPQ sediment, while rapid ureolysis was observed with additional yeast extract. This observation implies the successful stimulation of ureolytic bacteria native to Sellafield sediments using carbon sources is sediment and nutrient dependent. Urea plus Fe(III) (System E) for sediments RB23, RB27, and CR produced a more gradual pH increase and trend in urea degradation. Total urea decomposition in sediments CR and RB27 was accompanied by an increase in pH to 9.7. Incomplete ureolysis in sediment RB23 and PPQ produced an elevated pH of 8.7 and 7.7, respectively (Figure 1e).

### Strontium and Calcium Removal

**Figure 2:**
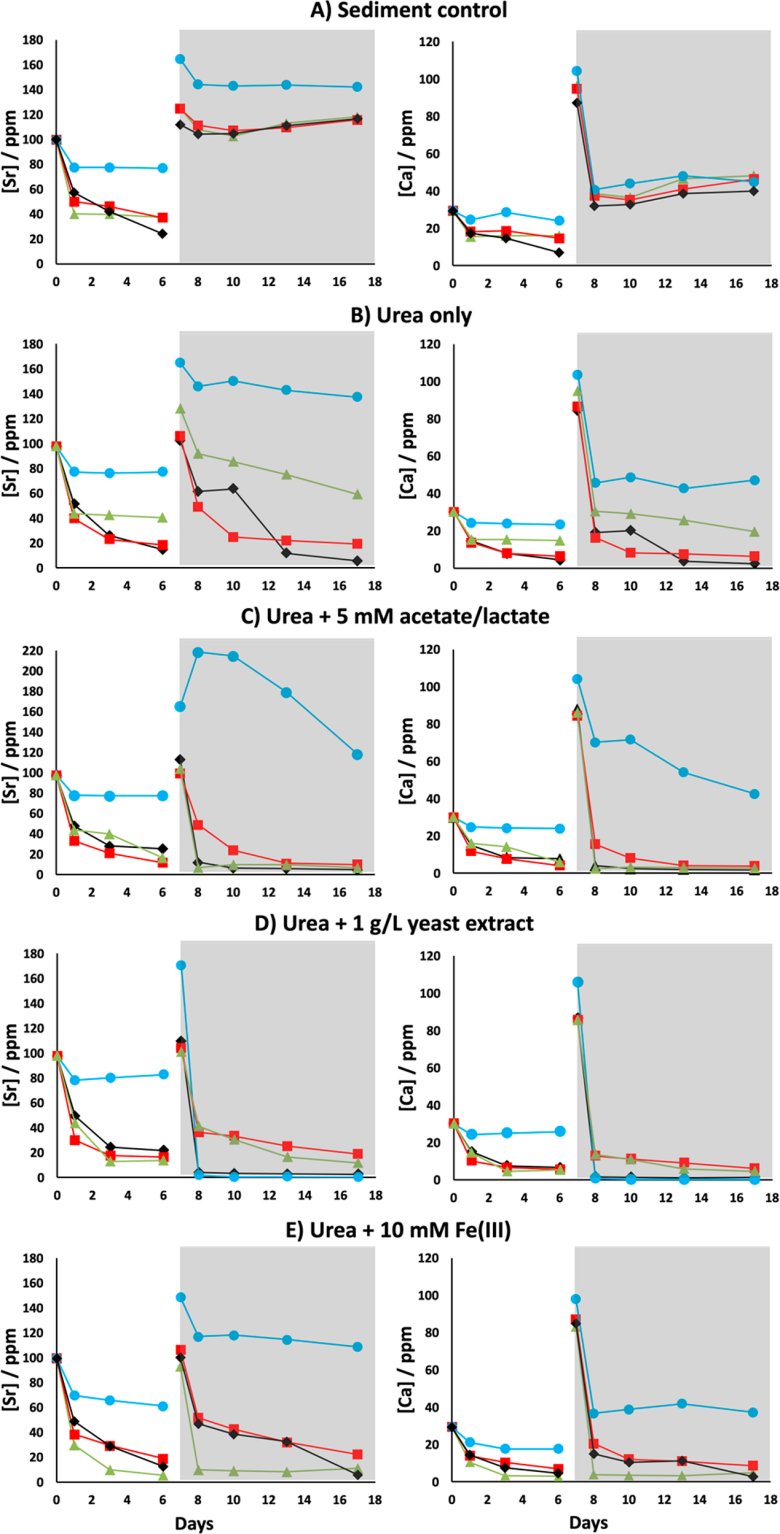
Strontium concentrations and corresponding Ca removal in Sellafield-relevant sediment microcosms. CR (black diamonds), PPQ (blue circles), RB23 (red squares), and RB27 (green triangles). Systems were spiked with 88 ppm of Sr^2+^ + 80 ppm of Ca^2+^ at day 7 to display clearer evidence of their enhanced concomitant removal with urea present. Phase 1 of Sr and Ca removal is represented by the blank background in individual plots, while Phase 2 is highlighted by the gray areas.

The PPQ sediment removed approximately 20% Sr after 1 day in the urea-free controls (System A). Sr removal in the urea-free controls for RB23, RB27, and CR sediments reached 40–60% by the same time point. Approximately 10% additional Sr was then removed following a spike of 88 ppm of Sr and 80 ppm of Ca added at day 7. Sr and Ca removal before and after this second Sr/Ca spike are represented as Phase 1 and Phase 2 in these experiments, respectively (Figure 2a). Generally, the urea-bearing incubations (Systems B to E) showed enhanced Sr removal by the end of Phase 1 for all sediments aside from PPQ, typically between 75% and 90% (Figure 2b–e). In urea-only incubations, Phase 1 saw Sr removal in sediments CR and RB23 of 85% and 82%, respectively. The presence of only urea failed to increase Sr removal during Phase 1 for sediment RB27; however, additional acetate and lactate enhanced Phase 1 strontium removal to 84%, which was similar to RB23 (87%) (Figure 2c). Amendments using yeast extract in addition to urea resulted in similar levels of Sr removal in RB23, RB27, and CR (78–87%) during Phase 1. For sediments CR and RB27, the presence of 10 mM Fe (III) in addition to urea generated the largest Phase 1 decrease in Sr of 88% and 94%, respectively.

Sr remediation was more variable between sediments during Phase 2 compared to Phase 1 in incubations only containing urea (Figure 2b). For this system, enhanced Sr and Ca removal continued in Phase 2 in all sediments, with the largest removal of Sr occurring in sediments RB23 and CR (82% and 94%, respectively) and lower levels of removal in sediments RB27 (54%) and PPQ (17%) (Figure 2b). With additional acetate and lactate, Phase 2 displayed near-complete Sr removal in sediments CR (94%), RB27 (93%), and RB23 (90%) and also enhanced Sr removal in the PPQ sediments (46%) (Figure 2c). The presence of yeast extract in addition to urea increased Sr removal in sediment RB23, RB27, and CR during Phase 2 to 82%, 88%, and 97% respectively. Most notably, additional yeast extract in the PPQ incubation produced the most significant removal of Sr observed in this study, achieving near-total Sr removal to subppm levels during Phase 2 (Figure 2d). With added 10 mM Fe(III), sediments RB23, RB27, and CR displayed a reduction in Sr of 79%, 89%, and 94%, respectively, during Phase 2 (Figure 2). For the PPQ sediment, this system produced only a 27% decrease in Sr during Phase 2. It is evident that PPQ sediment required additional carbon stimulation to enhance Sr removal in the presence of urea, which only appeared to occur during Phase 2, contrary to the other Sellafield sediments.

Strontium and calcium removal in the urea-free controls were attributed to adsorption (Figure 2a). The pH point of zero charge (pH_pzc_) of the silicates and oxides comprising the dominant mineralogy of the sediments ranges from approximately 2.5 to 7. At pH values > pH_pzc_, these mineral surfaces are negatively charged and favor the accumulation of adsorbed cations, e.g., Sr^2+^. This indicates that increasing pH above 7 will likely increase Sr adsorption and removal from solution, consistent with previous studies of Sr sorption to Sellafield-relevant sediments.^11^

Ureolysis by soil bacteria is not retarded under anaerobic conditions.^83^ Under anoxia, anaerobic bacteria may couple the enzymatic oxidation of simple organic acids (e.g., acetate and lactate) to the reduction of electron acceptors (e.g., Fe(III)) in order to conserve energy for growth; this has been demonstrated in previous studies for sediments RB23 and RB27.^71^ Increased urea degradation and more rapid evolution to high pH with amendments of acetate and lactate in sediments RB23, RB27, and CR compared to urea-only incubations was likely due to the enhanced growth of a ureolytic bacteria under anaerobic conditions (Figure 1b,c). In turn, this produced faster and more complete Sr and Ca removal during Phases 1 and 2 compared to urea-only systems (Figure 2b,c). Additional Fe(III) further enhanced the rate of ureolysis and concomitant pH increase in sediment RB27, which did not occur with the other sediments. This resulted in significantly more Sr removal relative to the urea-free control for RB27 and could be due to the stimulation of Fe(III)-reducing bacteria that could play a role in ureolysis. Yeast extract may also serve as an electron donor^84^ and is also commonly used as a micronutrient to help stimulate bacterial growth.^85^ Enhanced rates of ureolysis have been displayed in soils amended with yeast extract.^62,86^ A significant pH increase coincided with total urea degradation in all sediments amended further with yeast extract. For example, the RB23, RB27, and CR sediments amended with urea plus yeast extract reached pH 10 by day 3 and 6, respectively, after almost all of the urea has been decomposed (Figure 1d). The PPQ sediment similarly reached pH 10 on day 10 after the urea concentration had decreased by 50%, being the only PPQ system to observe both total urea degradation and an increase to pH ≥ 8.2 thereafter (Figure 1d). As with acetate and lactate amendments, it is likely the increases in pH are related to increased rates of ureolysis after stimulation with yeast extract, resulting in more rapid pH increases compared with the urea-only controls. Overall, amending Sellafield sediment microcosms with urea (and additional biostimulating compounds) correlated with increased and more rapid Sr and Ca removal, indicating that calcium carbonate precipitation may be occurring and responsible for enhancing Sr uptake. Microbially precipitated carbonates are well-known to simultaneously remove Ca^2+^ and Sr^2+^.^87−89^ Stimulating ureolytic bacteria with urea in pure culture studies has produced an increase in groundwater pH from circumneutral to ≥9 on the order of hours, under both oxic and anoxic conditions.^90,91^

### Environmental Scanning Emission Spectroscopy (ESEM)

**Figure 3:**
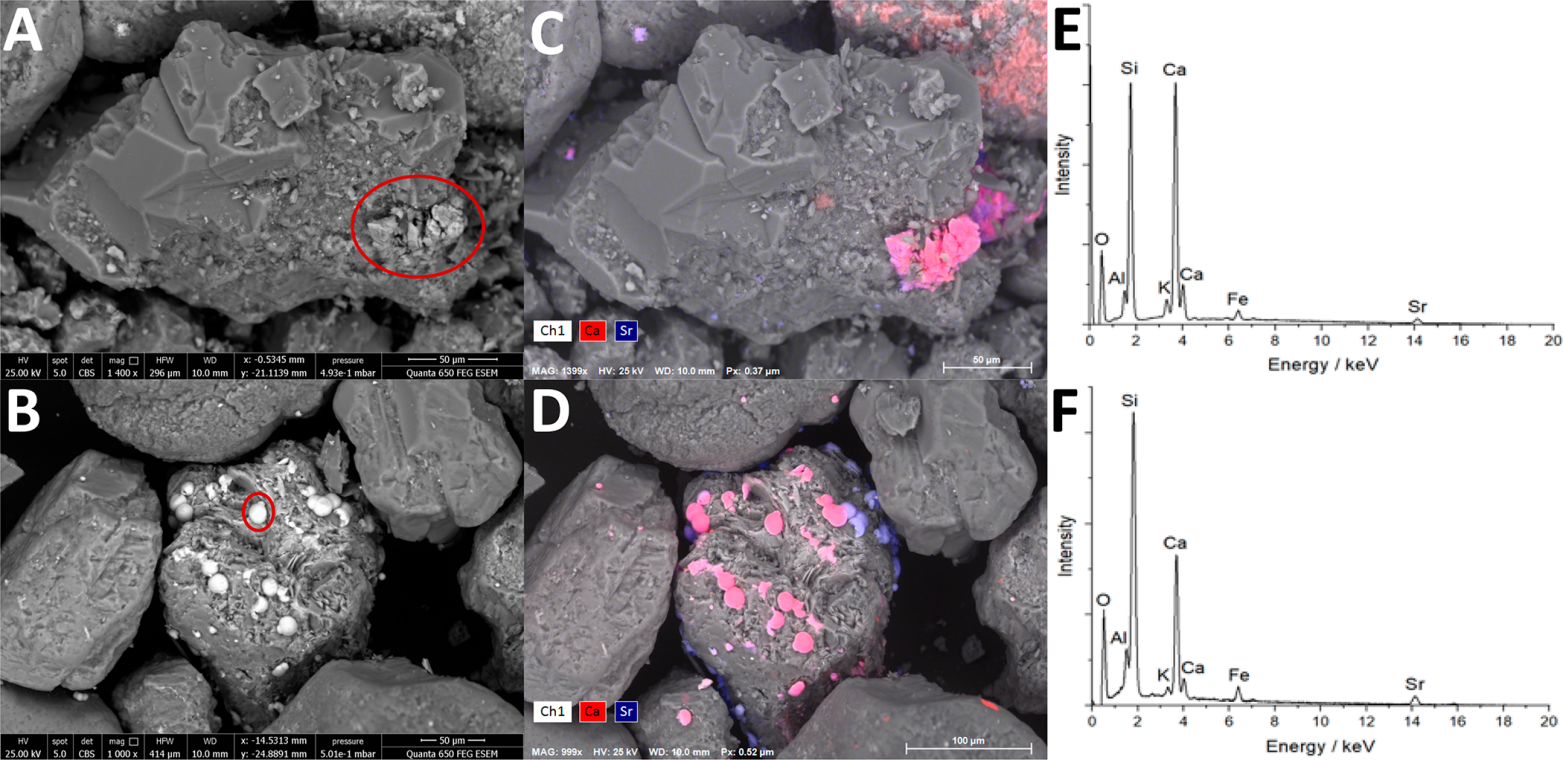
ESEM analysis of a rhombohedral precipitate formed in RB27 microcosm amended with 10 mM Fe(III) (a, c, and e) and a spheriodal precipitate formed in PPQ microcosm amended with 1 g/L yeast extract (b, d, and f), 17 days after incubation. (a and b) Images in backscatter mode. (c and d) Elemental maps displaying the correlation between Ca and Sr. (e and f) EDX spectra corresponding to the aggregate circled in red in the ESEM image.

Biostimulated sediments from RB27 amended with 10 mM Fe(III) and PPQ amended with 1 g/L yeast extract were analyzed using ESEM (Figure 3). Samples were predominantly composed of silicate (e.g., quartz and feldspar) grains coated with clay particles (e.g., chlorite and muscovite) approximately 100–200 μm in size. Backscatter imaging combined with energy dispersive spectroscopy (EDS) mapping revealed the presence of approximately 50 μm crystallites (Figure 3a,c) and spheroidal particles (Figure 3b,d) on the surface of the silicate grains. EDS analysis of these areas also indicates these particles are calcium-rich with strontium present (Figure 3e,f). The silicon, iron, and aluminum detected are due to the underlying silicate particles.

The morphology and composition of these particles indicates they are polymorphs of Sr-containing calcium carbonate, likely formed in the microcosms during the decomposition of urea.^92,93^ The precipitates formed in the RB27 system were more angular in appearance compared with the spheriodal particles observed in the PPQ sediment. Calcite tends to form rhombohedral crystals with distinct crystal faces,^94^ similar in appearance to the Ca/Sr-bearing particles present in the RB27 system (Figures 3a and S2), and other studies investigating Sr removal via urease-facilitated MICP.^95,96^ The spheriodal particles in the PPQ system suggests that vaterite is the calcium carbonate polymorph present, responsible for incorporating Sr during precipitation. A study by Sheng Han et al.^97^ identified micron-scale vaterite spherulites using SEM in a system where ammonia was used to increase pH from 8 to 11. During this process, high calcium carbonate supersaturation was achieved by rapidly dissolving CO_2_, which led to the formation of vaterite due to the sustained high supersaturation during heterogeneous nucleation.^98^

The RB27 system displayed a steady pH increase from 7.5 to 8.9 on days 1 to 6, coinciding with a near linear urea decomposition rate leading to calcite formation (Figure 1e). This corresponded to a decrease in strontium concentration from 30 to 6 ppm and concomitant calcium removal over the 5 day period (Figure 2e). In contrast, the PPQ system produced a much faster pH increase, rising from 7.5 to 9.6 on days 3 and 6, respectively (Figure 1d), leading to vaterite formation. Interestingly, in the PPQ system, the strontium concentration remained at ∼80 ppm between days 3 and 6 despite the marked pH increase, but decreased from 170 ppm on day 7 to below 1 ppm at day 8 (Figures 1d and 3d). The precipitation of vaterite has been shown to occur within minutes of supersaturation in both abiotic^43,97^ and pure culture^99,100^ systems and has the potential to uptake more Sr than calcite.^43^ The differing degrees of solution supersaturation (and the rate of supersaturation development) with respect to calcium carbonate in the PPQ and RB27 systems likely controlled the differences in Sr-containing calcium carbonate polymorphs precipitated.^101^

### PHREEQC

**Figure 4:**
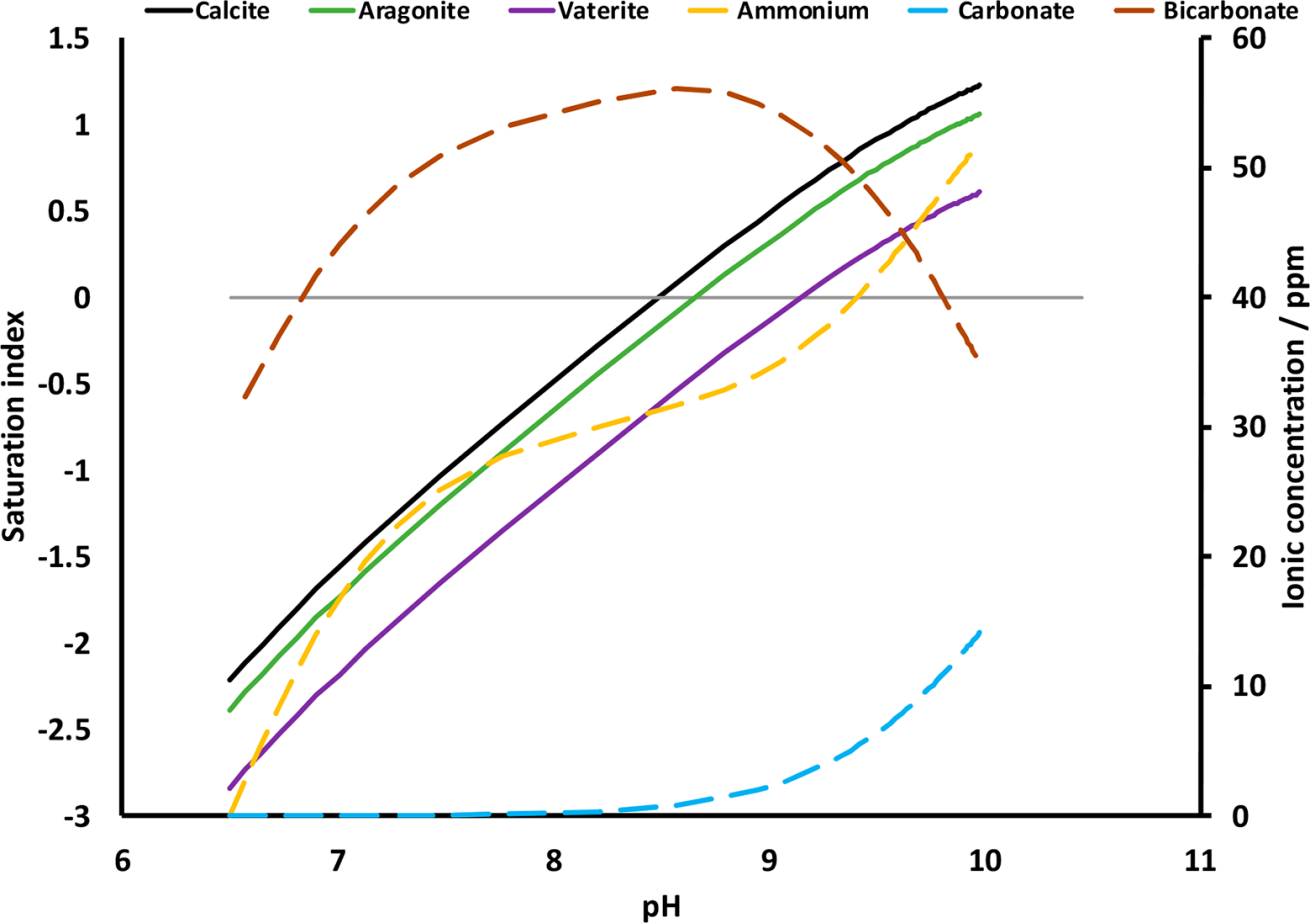
Selected PHREEQC output for the microbial hydrolysis of urea in strontium-bearing Sellafield groundwater, depicting the supersaturation of various Sr/Ca-containing carbonate phases.

In this study, a geochemical model simulating the impact of bacterial hydrolysis of urea in Sellafield-representative groundwater containing 100 ppm (∼1.14 mM) strontium was constructed using PHREEQC^79^ (Figures 4 and S3). Ureolysis and consequent geochemical changes were simulated over 1 day. The rate of urea hydrolysis (*k*_urea_) was assumed to follow the first order reaction  given the approximately linear trend in urea degradation for PPQ with 1 g/L yeast extract (between days 6 and 15) and RB27 with 10 mM Fe(III) (between days 1 and 25). A previous study investigating ureolytic MICP by indigenous microbial communities used a similar approximation to calculate the ureolysis rate constants.^59^ For the RB27 system, *k*_urea_ = 0.04 day^–1^, which was calculated for urea-stimulated natural groundwater supplemented with 1 g/L molasses.^59^ For the PPQ system, *k*_urea_ = 0.12 day^–1^, which was calculated for urea-stimulated artificial groundwater amended with a pure culture of *Sporosarcina pasteurii* (∼7.2 × 10^5^ cell/mL).^59^ There was no significant difference in modeled rate of increase in carbonate mineral supersaturation using *k*_urea_ = 0.04 day^–1^ (Figure 4) compared with 0.12 day^–1^. Model results showed an immediate pH increase, caused by the stoichiometric hydrolytic breakdown of 1 mol of urea to 2 mol of NH_4_^+^, generating alkalinity (Figure S4). As ureolysis progresses, several calcium carbonate minerals (e.g., calcite and vaterite) became oversaturated within the pH range observed in this study (from pH 6.5 to 10). Clearly, the simulation suggests that the ureolysis is capable of altering Sellafield groundwater geochemistry to produce conditions conducive to carbonate mineral formation. For the RB27 system, an increase in pH from 7.5 to 8.1 to 8.9 was observed from days 1, 3, and 6, respectively (Figure 1e). The model suggests that Sellafield AGW is undersaturated with respect to calcium carbonate at pH 7.5 (Figure 4) but shows an increase in the saturation index (SI) for calcite from −1.04 to 0.44 as pH increases from 7.5 to 8.9. Conversely, the PPQ system displayed a significant increase from pH 7.5 on day 3 to pH 9.6 on day 6 (Figure 1d). The model suggests this rapid evolution of high pH and alkalinity leaves the solution oversaturated with respect to vaterite (SI = 0.36).

In the urea-free controls, RB27 removed 50% more Sr than PPQ via adsorption (Figure 2a). Additionally, the rate of ureolysis and the development of sufficiently high pH and alkalinity necessary for calcium carbonate precipitation was much slower in the RB27 system with 10 mM Fe(III) than the PPQ system with 1 g/L yeast extract (Figures 1e, 4, and S4). Thus, these factors likely contributed to lowering the degree of calcium carbonate supersaturation in the RB27 system, favoring slower and surface-mediated growth of strontium-containing calcite. In contrast, in the PPQ system, rapid pH and alkalinity increases likely pushed the Sellafield AGW much further out of equilibrium with respect to Ca, producing a far higher degree of supersaturation and likely facilitating nucleation-dominated vaterite precipitation (Figures 1d, 4, and S4).

### X-ray Absorption Spectroscopy

**Figure 5:**
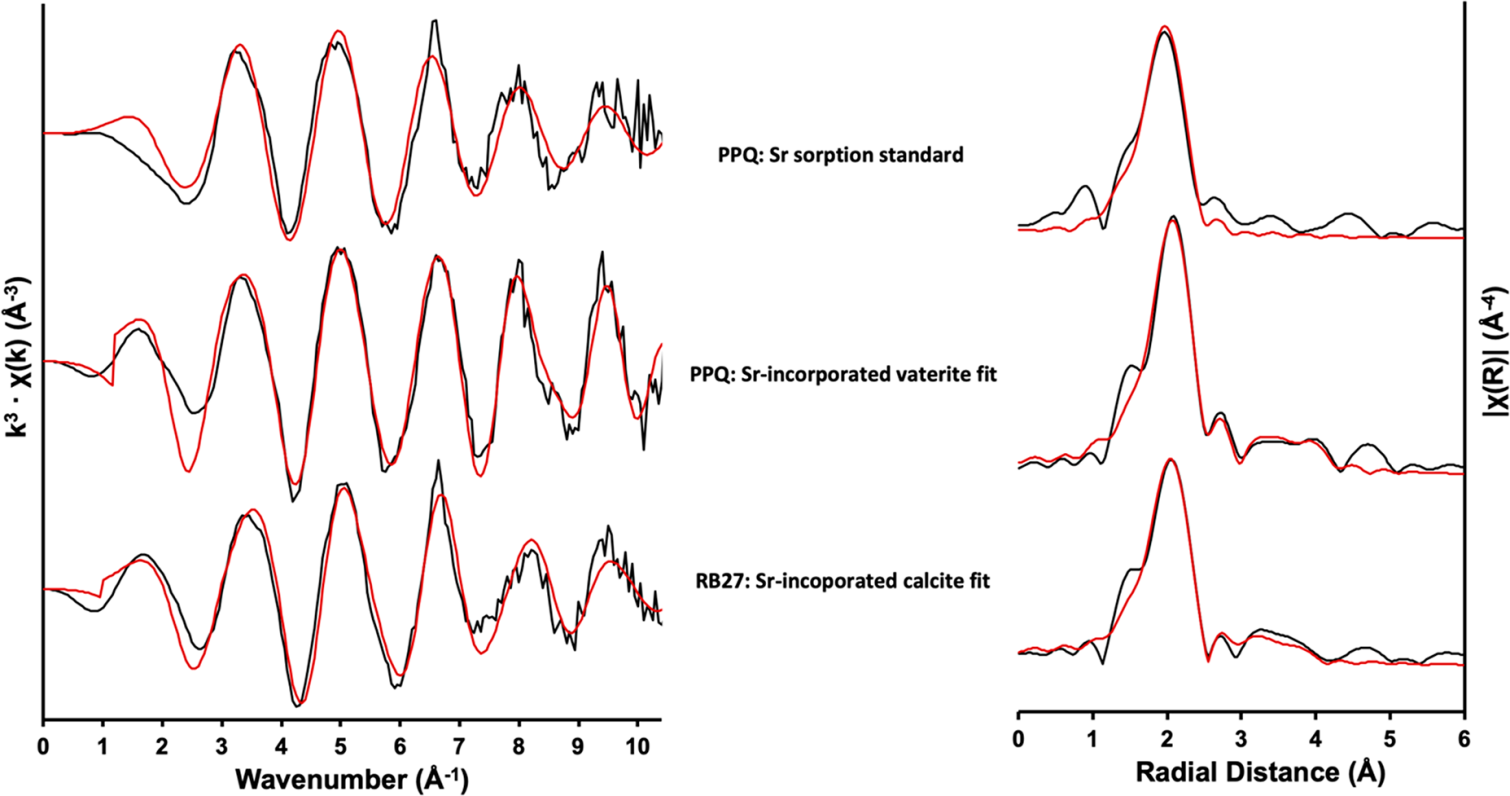
Background-subtracted Sr *K*-edge EXAFS data (left-hand side) and corresponding Fourier transformations (right-hand side) obtained on biostimulated sediments from PPQ amended with 1 g/L yeast extract and RB27 amended with 10 mM Fe(III). A description of the strontium-associated calcium carbonate phase used to fit the experimental data is also provided. Experimental data = black line, fit = red line.

The speciation of Sr within RB27 sediments stimulated with urea and 10 mM Fe(III) (E) and PPQ sediments stimulated with urea and 1 g/L yeast extract (D) was analyzed via Sr *K*-edge XAS spectroscopy of the microcosm end products (Figure 5). Given that the previous modeling, aqueous geochemical, and microscopic analyses suggested the potential association of Sr^2+^ with CaCO_3_ mineralization, the local environment of various Sr^2+^-incorporated calcium carbonate phases was used to inform the fits to the spectra.^43,47,52,102,103^

The untreated PPQ system returned a spectrum that was best fit with 9 O atoms at 2.6 Å indicative of outer-sphere adsorption.^82,104,105^ The best fit to the spectrum from the PPQ sample indicated a central Sr atom in 8-fold coordination (8 O atoms at 2.53 Å) with an addition of 4 C atoms at 2.95 Å. The model also includes 2 distinct Sr–Ca shells at 3.95 and 4.21 Å. However, substituting the 2 Ca atoms at 4.21 Å with Sr atoms produced an almost identical fit. Splitting the Sr–Ca coordination path into 2 shells improved the fit with statistical significance compared with adding 4 Ca atoms at 4.05 Å (Table S1). Overall, this coordination environment is indicative of Sr substitution for Ca within vaterite.^43,47,103,106^ The best fit achieved here was in closer agreement to that of Littlewood et al.,^43^ who studied Sr-incorporated vaterite from a 0.1% Sr^2+^ solution (Table 2). The only notable difference in shell fitting presented here compared with that presented in Littlewood et al.^43^ is the splitting of the distal Ca shell with slightly different Sr–Ca distances. However, we note that the Debye–Waller factors for the single Sr–Ca shell in the Littlewood et al.^43^ study is significantly higher than those of the two individual Sr–Ca shells fitted here (0.014 vs 0.004 and 0.007). This indicates that there may be significant structural disorder associated with the single Sr–Ca shell in the current study, with the Ca atoms spread over the range of distances. This is broadly consistent with the split Sr–Ca shells in this study, with improvements in data quality and *k*-range a likely reason for the resolution of 2 Ca shells in this study. In the literature, Ca *K*-edge spectra for vaterite often varies considerably,^107,108^ referred to without experimental data,^53,54^ not modeled at *R* > 3 Å^103^ or at all,^109^ not accompanied by model fitting parameters,^110^ or are fit poorly/only in conjunction with the “conventional” 6 Ca atoms at approximately 4.2 Å (in accordance with ref (111)).^55,112^ Splitting the Ca shell at approximately 3.9–4.3 Å has not been conducted in studies analyzing the structure of vaterite using Ca *K*-edge. Instead, a Ca shell at 3.95 Å has been reported for calcite^54^ and aragonite.^53^ In addition, the presence of structural disorder of vaterite crystals formed under environmental conditions is widely accepted^102^ and, in the present study, it is likely enhanced by the presence of Sr^2+^ ions substituting for smaller Ca^2+^ ions (1.18 and 1.00 Å, respectively).^20^ This distortion likely explains the presence of the split distal Sr–Ca shell in two with similar Sr–Ca distances.

**Table 2:** Details of EXAFS Fitting Parameters for the Sr Biominerals Formed in the Respective PPQ and RB27 Systems^a^

sediment/system; fit description	coordination path	coordination number	coordination number^43^	*R* (Å)	*R* (Å)^43^	σ^2^ (Å^2^)	σ^2^ (Å^2^)^43^
PPQ	Sr–O	9		2.60		0.012	
Sr sorption standard							
PPQ + 1 g/L yeast extract	Sr–O	8	8	2.53	2.52	0.010	0.011
Sr-incorporated vaterite	Sr–C	4	4	2.95	3.10	0.006	0.025
	Sr–Ca	2		3.95		0.007	
	Sr–Ca	2	4	4.21	4.19	0.004	0.014
RB27 + 10 mM Fe (III)	Sr–O	9	9	2.60	2.57	0.011	0.017
Sr-incorporated calcite	Sr–C	5	5	3.03	3.10	0.030	0.020
	Sr–Ca	4	4	4.10	4.12	0.019	0.018

a *R* = atomic distance; σ^2^ = Debye–Waller factor. The amplitude factor (S02) was set to 1 for each sample.

As postulated above, spherulitic Ca-bearing precipitates evident within the PPQ system likely signify vaterite formation (Figures 3b,d). Results from the PHREEQC calculation of the ureolytic AGW system indicated vaterite supersaturation is achieved at pH ∼ 9.1, which was observed in this system during the rapid pH increase. This is consistent with batch experiments investigating Sr bioremediation via urea-induced MICP using pure cultures of Sr-resistant *Halomonas* sp.^113^ and *Bacillus pasteurii*,^58,114^ which successfully remediated Sr through carbonate-phase formation, including vaterite.

Vaterite is generally considered an unstable intermediate that nucleates rapidly from amorphous calcium carbonate, eventually transforming to calcite within hours.^43,102,115,116^ This study suggests that Sr-incorporated vaterite may describe a more stable Sr-incorporated phase than first conceived under environmentally relevant conditions. Sustained ammonium generation, inducing a consistent elevated pH, and the consequent retention of a high degree of supersaturation might explain the persistence of vaterite.^97^ The transformation of vaterite to more stable calcium carbonate polymorphs such as calcite could reduce the partitioning of Sr.^117^ However, the retention of Sr during calcium carbonate recrystallization is complicated by other factors (especially under environmental conditions) including the amount of initial vaterite precipitation (mediated by the initial ionic strength of a solution) and the rate of vaterite dissolution which, in turn, partially regulates the rate of calcite precipitation.^43^

Fitting the EXAFS spectrum for RB27 using a crystal structure for Sr^2+^-incorporated calcite provided an excellent fit, detailing a central Sr^2+^ atom coordinated to 9 O atoms at 2.60 Å, 5 C atoms at 3.03 Å, and 4 Ca atoms at 4.10 Å (Figure 5 and Table 2). The model contained near identical interatomic distances and shell occupancies to those for the chemical precipitation of calcium carbonate in strontium-rich systems.^43^ The lack of a distinct doublet at ∼16 115 eV in either XANES spectrum suggested the absence of 6-fold coordination indicative of Sr-incorporated calcite (Figure S5).^37,50,118,119^ The occurrence of a single peak indicates Sr^2+^ in 8/9 fold coordination, which is consistent with the EXAFS fit. Elevated strontium concentrations present in these systems likely results in Sr^2+^ occupying lattice sites alternative to the position of 6-fold-coordinated Ca^2+^ ions when coprecipitated with calcium carbonate, as reported by Littlewood et al.^43^ This likely occurs due to the higher concentrations of Sr^2+^ within the calcite crystal. Overall, elucidation of the two calcium carbonate polymorphs highlights the differing degrees of supersaturation achieved in these systems. More Sr was removed in the PPQ system, attributed to vaterite coprecipitation at a higher Sr^2+^ concentration.

### Microbial Community Analysis

**Figure 6:**
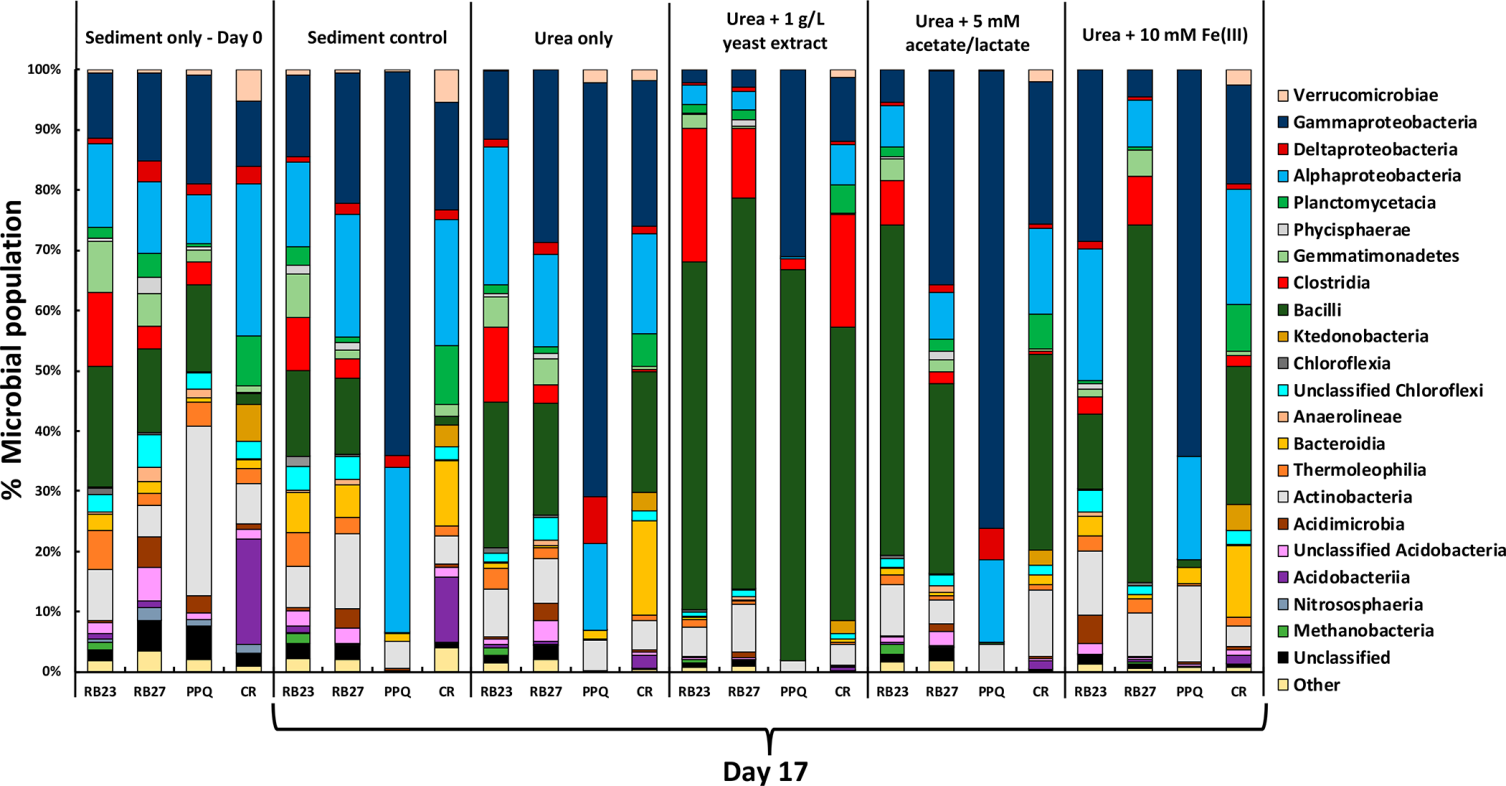
Bacterial phylogenetic diversity within the Sellafield-related sediments before and 17 days after biostimualtion with urea and other compounds aimed at promoting ureolytic conditions. The displayed classes account for greater than 1% of the microbial community.

DNA extractions were performed on various sediment samples obtained on days 0 and 17, and the 16S rRNA genes amplified and sequenced to help identify changes in the microbial communities within the urea-free controls and urea-stimulated sediments. A diverse indigenous community was identified in each sediment type prior to experimental incubations (Figure 6). For each system and sediment, a diverse range of bacteria was maintained after 17 days. Adding solely urea to sediments produced a slight shift toward a microbial community associated with ureolysis. Several *Bacillus* species, belonging to the class Bacilli, are well-documented as being capable of hydrolyzing urea.^120−123^ When compared to urea-free controls after 17 days, urea-only amendments enriched the proportion of Bacilli from 14% to 24% in RB23, 1% to 20% in CR, and 13% to 19% in RB27, while also enhancing Sr and Ca removal. However, microcosms modified with additional biostimulating compounds and urea produced the greatest shifts in microbial community structure.

Species of the class Bacilli constituted 14% of the PPQ sediment prior to incubations. Incubating PPQ with yeast extract in addition to urea was the only PPQ system to enrich species belonging to the microbial class Bacilli (65%) (Figure 6). The other PPQ incubations failed to maintain a relative proportion of Bacilli >1%. In the PPQ system amended with yeast extract, the closest known relatives assigned to two OTUs (operational taxonomic units), comprising 25% of the microbial population, were strains most closely related to *Sporosarcina pasteurii*,^124,125^ previously known as *Bacillus pasteurii*. The ureolytic precipitation of calcite has been widely demonstrated using pure cultures of *Sporosarcina pastuerii*,^126,127^ including studies that successfully coprecipitated Sr with calcite.^67,96^ While biostimulations using yeast extract have been shown to increase the population of *Bacillus* species in soil from 5% to 85–99%,^128^*Bacillus* urease has also precipitated spheroidal vaterite after increasing the pH of the system from 6.8 to 9.^99^ Incubating urea-stimulated PPQ sediments with yeast extract yielded the only PPQ system with enhanced Sr/Ca removal compared to urea-free controls. It also remained the only PPQ incubation to raise the groundwater pH above 8.2, consistent with complete urea degradation. Thus, results of the DNA extraction support geochemical measurements displaying that yeast extract amendments played a crucial role in enhancing ureolysis in PPQ sediment and subsequent Sr remediation. An OTU assigned to the urease-positive species *Methylophilus methylotrophus* constituted 8.5% of the PPQ urea-free control population,^129^ enriched to 29.4% in urea-only incubations (Table S2). However, amending PPQ solely with urea failed to stimulate urea decomposition or enhance Sr/Ca removal. The dominance of Bacilli species in the yeast-amended PPQ system, as well as other biostimulated sediments, appears consistent with the development of ureolytic conditions necessary for increasing Sr/Ca removal compared with urea-free controls.

Species of Bacilli constituted 14% of the microbial population indigenous to RB27 both at the start and 17 days after urea-free controls were constructed (Figure 6). Incubating RB27 with urea and an additional 10 mM Fe(III) for 17 days led to the prevalence of Bacilli (60%), comparable to further additions of yeast extracted (65%) and far greater than enrichments produced by urea only (19%) and 5 mM acetate/lactate (30%) amendments (Table S3). The five most abundant OTUs by day 17 all belonged to the class Bacilli. Three of the five OTUs were assigned to ureolytic species of *Sporosarcina* and constituted around 20% of the total microbial population. The closest known relatives of the three OTUs included a facultatively anaerobic strain of *Sporosarcina aquimarina* (13.2%)^130,131^ as well as *Sporosarcina pasteurii* (4.1%)^59,124^ and *Sporosarcina ginsengoli* (5%).^132^

### Fe(II) Generation Facilitated by Bacterial Ureolysis

**Figure 7:**
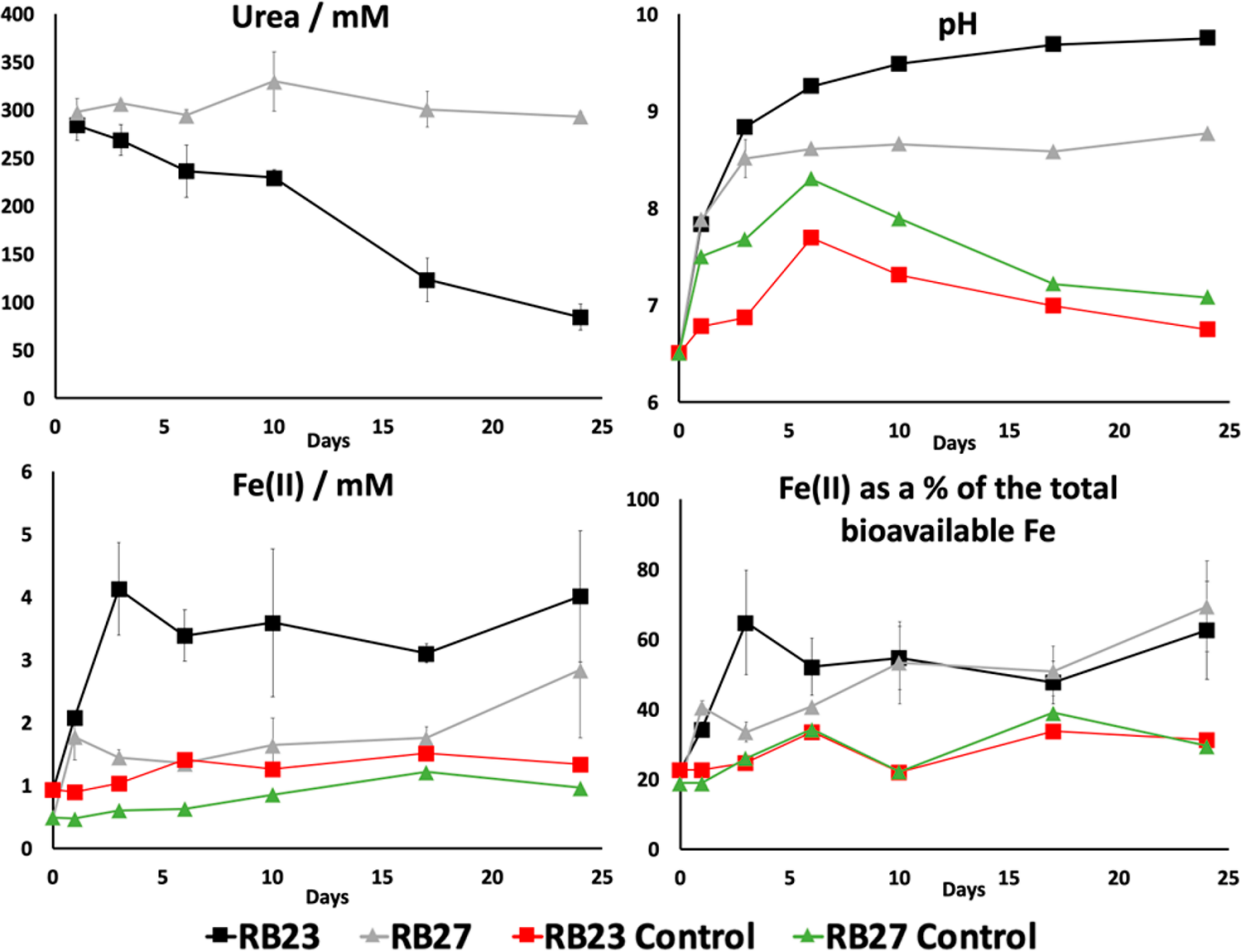
Microbial Fe(III) reduction in Sellafield sediment microcosms. RB23 urea only (black squares), RB27 urea only (gray triangles), RB23 control (red squares), and RB27 control (green triangles).

The reduction of bioavailable Fe(III) to Fe(II) within Sellafield sediments RB23 and RB27 was enhanced with the addition of urea (Figure 7). In both sediments, the percentage of bioavailable Fe as Fe(II) increased from approximately 20–60% after 25 days of anaerobic incubation with solely urea. Parallel urea-free controls constructed using only sediments (with no amendments) displayed an increase in Fe(II) from approximately 20% to 30%.

Ureolysis-generated ammonium may have served as an electron donor in generating microbial reduction of Fe(III). Anammox (anaerobic ammonium oxidation) processes play a major role in global nitrogen cycling within aquifers, particularly nitrogen loss.^133^ In a separate process, certain organisms are able to couple the oxidation of ammonium to Fe(III) reduction during anaerobic respiration,^134^ a process termed “feammox” that has been observed in Fe(III)-rich anoxic soils [eq 5^135^].
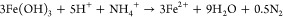
5

Bacteria capable of enzymatically reducing Fe(III) to Fe(II) are often similarly capable of directly reducing U(VI) to U(IV)^136^ while microbial Fe(II) generation is capable of indirectly reducing U(VI) to U(IV),^137^ Tc(VII) to Tc(IV),^82,138^ and Np(V) to Np(IV)^139^ with the reduced forms typically poorly soluble compared to the oxidized species. Thus, results from this study indicate that the ureolytic stimulation of bacteria indigenous to Sellafield sediments could be used to immobilize both strontium and uranium/neptunium/technetium through MICP and feammox processes respectively, in a coupled system.

### Conclusion and Environmental Significance

While MICP by urease encoding bacteria have been investigated in sediments from other contaminated nuclear sites, this represents the first study of urea-facilitated MICP in Sellafield sediments and also quantifies the impact of added electron donors/nutrients (i.e., acetate, lactate, and yeast extract) and electron acceptors (i.e., Fe(III)). To the best of our knowledge, this is the first time that enhanced strontium removal by urea-facilitated MICP has been demonstrated by indigenous sediment microbial communities. The rate and extent of concomitant Sr^2+^ and Ca^2+^ removal at elevated pH varied between the sediments, and the biostimulation regime applied. Maximal removal of Sr (170 ppm to subppm concentrations over the course of 1 day) was noted in the PPQ sediment microcosm amended with urea and yeast extract. This system reached approximately pH 10 before extensive Sr removal. Spherical Ca/Sr-bearing precipitates identified in the remediated PPQ sediments using ESEM and corresponding EDX analyses suggested that Sr coprecipitated with the calcium carbonate polymorph vaterite. EXAFS analysis of the sediments further supported the hypothesis that the end point of Sr remediation was indeed Sr-incorporated vaterite. The RB27 system amended with urea and additional Fe(III) reached a final pH > 9.5, coinciding with a reduction in strontium concentration from 93 to 10 ppm over 1 day. Analyzing the remediated sediments in the RB27 system using EXAFS and ESEM suggested that Sr was immobilized in calcite. While calcite is a more stable polymorph of calcium carbonate, vaterite precipitates more rapidly and is capable of incorporating more Sr.^43^ Thus, in addition to the sediment characteristics, the time scale and aqueous strontium concentrations are important considerations for perspective *in situ* remediation strategies when targeting strontium removal as various Sr-associated carbonate phases. Anaerobic microcosms using Sellafield sediments RB23 and RB27 also showed an unexpected increase in microbial Fe(III) with urea added. Enhanced Fe(III) bioreduction coupled to the oxidation of NH_4_^+^ formed during ureolysis may have been responsible for enhancing Fe(II) production in these systems. Accumulating sufficient NH_4_^+^ from ureolysis may, therefore, enhance further beneficial microbial activity for the remediation of redox-active radionuclide species such as U, Np, and Tc. This work has significant implications for the remediation of ^90^Sr at Sellafield through MICP, in addition to the potential cleanup of redox active radionuclides. The need for further work to define the utility of these processes at a larger scale using flow-through systems more representative of the dynamic natural subsurface in order to assess the large-scale application and long-term performance of this technology is clear.

## Supporting Information Available

Additional information including a description of the extraction procedure for molecular ecology analysis, XRD analysis of certain sediments, EXAFS data and fitting parameters, and microbial diversity analysis; figures displaying XRD analysis of certain sediments, ESEM images of a Ca/Sr-bearing precipitate formed in this study, PHREEQC input and output files, and XANES spectra (PDF)

## References

[ref1] Vermeul V. R.; Szecsody J. E.; Fritz B. G.; Williams M. D.; Moore R. C.; Fruchter J. S. An Injectable Apatite Permeable Reactive Barrier for In Situ 90Sr Immobilization. Groundw Monit Remediat. 2014, 34 (2), 28–41. 10.1111/gwmr.12055.

[ref2] Peterson R. E.; Poston T. M. Strontium-90 at the Hanford Site and Its Ecological Implications; Pacific Northwest Nuclear Laboratory: Richland, WA, 2000.

[ref3] Zachara J. M.; Serne J.; Freshley M.; et al. Geochemical Processes Controlling Migration of Tank Wastes in Hanford’s Vadose Zone. Vadose Zo J. 2007, 6 (4), 985–1003. 10.2136/vzj2006.0180.

[ref4] Romanovskiy V. N.; Smirnov I. V.; Babain V. A.; et al. The universal solvent extraction (unex) process. i. development of the unex process solvent for the separation of cesiumstrontium and the actinides from acidic radioactive waste. Solvent Extr. Ion Exch. 2001, 19 (1), 1–21. 10.1081/SEI-100001370.

[ref5] Tolstykh E. I.; Degteva M. O.; Peremyslova L. M.; et al. Reconstruction of long-lived radionuclide intakes for techa riverside residents: Strontium-90. Health Phys. 2011, 101 (1), 28–47. 10.1097/HP.0b013e318206d0ff. 21617390

[ref6] Stefanovsky S.; Yudintsev S.; Gieré R.; Lumpkin G. Nuclear waste forms. Geol Soc. London, Spec Publ. 2004, 236, 37–63. 10.1144/GSL.SP.2004.236.01.04.

[ref7] Sylvester P.; Clearfield A. The removal of strontium and cesium from simulated hanford groundwater using inorganic ion exchange materials. Solvent Extr. Ion Exch. 1998, 16 (6), 1527–1539. 10.1080/07366299808934593.

[ref8] Hartman M. J.; Morasch L. F.; Webber W. D. Hanford Site Groundwater Monitoring for Fiscal Year 2005; Pacific Northwest National Laboratory, 2006.

[ref9] Christensen G. C.; Romanov G. N.; Strand P.; et al. Radioactive contamination in the environment of the nuclear enterprise “Mayak” PA. Results from the joint Russian-Norwegian field work in 1994. Sci. Total Environ. 1997, 202 (1–3), 237–248. 10.1016/S0048-9697(97)00119-8. 9241887

[ref10] Shagina N. B.; Vorobiova M. I.; Degteva M. O.; et al. Reconstruction of the contamination of the Techa River in 1949–1951 as a result of releases from the MAYAK Production Association. Radiat Environ. Biophys. 2012, 51 (4), 349–366. 10.1007/s00411-012-0414-0. 22797860

[ref11] Wallace S. H.; Shaw S.; Morris K.; Small J. S.; Fuller A. J.; Burke I. T. Effect of groundwater pH and ionic strength on strontium sorption in aquifer sediments : Implications for 90 Sr mobility at contaminated nuclear sites. Appl. Geochem. 2012, 27, 1482–1491. 10.1016/j.apgeochem.2012.04.007.

[ref12] Steinhauser G.; Brandl A.; Johnson T. E. Comparison of the Chernobyl and Fukushima nuclear accidents: a review of the environmental impacts. Sci. Total Environ. 2014, 470, 800–817. 10.1016/j.scitotenv.2013.10.029. 24189103

[ref13] Oikawa S.; Takata H.; Watabe T.; Suzuki C.; Nakahara M.; Misonoo J. Long term temporal changes of 90 Sr and 137 Cs in seawater, bottom sediment and marine organism samples. From the Chernobyl accident to immediately after the Fukushima accident. Bunseki Kagaku (Japan Anal. 2013, 62 (6), 455–474. 10.2116/bunsekikagaku.62.455.

[ref14] Matsunaga T.; Ueno T.; Amano H.; et al. Characteristics of Chernobyl-derived radionuclides in particulate form in surface waters in the exclusion zone around the Chernobyl Nuclear Power Plant. J. Contam Hydrol. 1998, 35 (1–3), 101–113. 10.1016/S0169-7722(98)00119-3.

[ref15] Casacuberta N.; Masqué P.; Garcia-Orellana J.; Garcia-Tenorio R.; Buesseler K. O. 90Sr and 89Sr in seawater off Japan as a consequence of the Fukushima Dai-ichi nuclear accident. Biogeosciences. 2013, 10 (6), 3649–3659. 10.5194/bg-10-3649-2013.

[ref16] Konno M.; Takagai Y. Determination and Comparison of the Strontium-90 Concentrations in Topsoil of Fukushima Prefecture before and after the Fukushima Daiichi Nuclear Accident. ACS Omega. 2018, 3 (12), 18028–18038. 10.1021/acsomega.8b02640.

[ref17] Ohno T.; Hirono M.; Kakuta S.; Sakata S. Determination of strontium 90 in environmental samples by triple quadrupole ICP-MS and its application to Fukushima soil samples. J. Anal At Spectrom. 2018, 33 (6), 1081–1085. 10.1039/C8JA00017D.

[ref18] Kashparov V. A.; Lundin S. M.; Khomutinin Y. V.; et al. Soil contamination with 90Sr in the near zone of the Chernobyl accident. J. Environ. Radioact. 2001, 56 (3), 285–298. 10.1016/S0265-931X(00)00207-1. 11468820

[ref19] Whittaker E. J. W.; Muntus R. Ionic radii for use in geochimstry. Geochim. Cosmochim. Acta 1970, 34, 945–956. 10.1016/0016-7037(70)90077-3.

[ref20] Shannon R. D. Revised Effective Ionic Radii and Systematic Studies of Interatomic Distances in Halides and Chalcogenides. Acta Crystallogr. Sect A Cryst. physics, diffraction, Theor Gen Crystallogr. 1976, 32 (5), 751–767. 10.1107/S0567739476001551.

[ref21] Erten H.; Aksoyoglu S.; Hatipoglu S.; Gokturk H. Sorption of Cesium and Strontium on Montmorillonite and Kaolinite. Radiochim Acta. 1988, 44, 147–151. 10.1524/ract.1988.4445.1.147.

[ref22] Dyer A.; Chow J. K. K.; Umar I. M. The uptake of caesium and strontium radioisotopes onto clays. J. Mater. Chem. 2000, 10 (12), 2734–2740. 10.1039/b006662l.

[ref23] Sahai N.; Carroll S. A.; Roberts S.; O’Day P. A. X-ray absorption spectroscopy of strontium (II) coordination: II. Sorption and precipitation at kaolinite, amorphous silica, and goethite surfaces. J. Colloid Interface Sci. 2000, 222 (2), 198–212. 10.1006/jcis.1999.6562. 10662515

[ref24] Norden B. M.; Ephraim J. H.; Allard B. The influence of a fulvic acid on the adsorption of europium and strontium by alumina and quartz: Effects of ph and ionic strength. Radiochim Acta. 1994, 65 (4), 265–270. 10.1524/ract.1994.65.4.265.

[ref25] Nie Z.; Finck N.; Heberling F.; Pruessmann T.; Liu C.; Lützenkirchen J. Adsorption of Selenium and Strontium on Goethite: EXAFS Study and Surface Complexation Modeling of the Ternary Systems. Environ. Sci. Technol. 2017, 51 (7), 3751–3758. 10.1021/acs.est.6b06104. 28285518

[ref26] Hongxia Z.; Xiaoyun W.; Honghong L.; Tianshe T.; Wangsuo W. Adsorption behavior of Th(IV) onto Illite: Effect of contact time, pH value, ionic strength, humic acid and temperature. Appl. Clay Sci. 2016, 127–128, 35–43. 10.1016/j.clay.2016.03.038.

[ref27] Gu B.; Doner H. E. Adsorption of hydroxy-Al polycations and destabilization of Illite and montmorillonite suspensions. Clays Clay Miner. 1990, 38 (5), 493–500. 10.1346/CCMN.1990.0380505.

[ref28] Nosrati A.; Addai-Mensah J.; Skinner W. Influence of mineral chemistry on electrokinetic and rheological behavior of aqueous muscovite dispersions. Ind. Eng. Chem. Res. 2011, 50 (19), 11087–11096. 10.1021/ie101548f.

[ref29] Kosmulski M. pH-dependent surface charging and points of zero charge. IV. Update and new approach. J. Colloid Interface Sci. 2009, 337 (2), 439–448. 10.1016/j.jcis.2009.04.072. 19501839

[ref30] Kosmulski M. Compilation of PZC and IEP of sparingly soluble metal oxides and hydroxides from literature. Adv. Colloid Interface Sci. 2009, 152 (1–2), 14–25. 10.1016/j.cis.2009.08.003. 19744641

[ref31] Trivedi P.; Axe L. A comparison of strontium sorption to hydrous aluminum, iron, and manganese oxides. J. Colloid Interface Sci. 1999, 218 (2), 554–563. 10.1006/jcis.1999.6465. 10502389

[ref32] Small T. D.; Warren L. A.; Ferris F. G. Influence of ionic strength on strontium sorption to bacteria, Fe(III) oxide, and composite bacteria-Fe(III) oxide surfaces. Appl. Geochem. 2001, 16 (7–8), 939–946. 10.1016/S0883-2927(00)00065-2.

[ref34] Istok J. D.; Senko J. M.; Krumholz L. R.; et al. In Situ Bioreduction of Technetium and Uranium in a Nitrate-Contaminated Aquifer. Environ. Sci. Technol. 2004, 38 (2), 468–475. 10.1021/es034639p. 14750721

[ref35] Sellafield Ltd. Monitoring Our Environment: Discharges and Environmental Monitoring. Annual Report 2021; 2022.

[ref37] Pingitore N. E.; Lytle F. W.; Davies B. M.; Eastman M. P.; Eller P. G.; Larson E. M. Mode of incorporation of Sr2+ in calcite: Determination by X-ray absorption spectroscopy. Geochim. Cosmochim. Acta 1992, 56 (4), 1531–1538. 10.1016/0016-7037(92)90222-5.

[ref38] Kinsman D. J. J.; Holland H. D. The co-precipitation of cations with CaCO3—IV. The co-precipitation of Sr2+ with aragonite between 16° and 96° C. Geochim. Cosmochim. Acta 1969, 33 (1), 1–17. 10.1016/0016-7037(69)90089-1.

[ref39] Chesney E. J.; McKee B. M.; Blanchard T.; Chan L. H. Chemistry of otoliths from juvenile menhaden*Brevoortia patronus*: Evaluating strontium, strontium:calcium and strontium isotope ratios as environmental indicators. Mar. Ecol.: Prog. Ser. 1998, 171, 261–273. 10.3354/meps171261.

[ref40] Brown R.; Severin K. P. Elemental distribution within polymorphic inconnu (*Stenodus leucicthys*) otoliths is affected by crystal structure. Can. J. Fish Aquat Sci. 1999, 56, 1898–1903. 10.1139/f99-127.

[ref41] Holland H. D.; Kirsipu T. V.; Huebner J. S.; Oxburgh U. M. On Some Aspects of the Chemical Evolution of Cave Waters. J. Geol. 1964, 72 (1), 36–67. 10.1086/626964.

[ref42] Pingitore N. E.; Eastman M. P. The coprecipitation of Sr2+ with calcite at 25°C and 1 atm. Geochim. Cosmochim. Acta 1986, 50 (10), 2195–2203. 10.1016/0016-7037(86)90074-8.

[ref43] Littlewood J. L.; Shaw S.; Peacock C. L.; Bots P.; Trivedi D.; Burke I. T. Mechanism of Enhanced Strontium Uptake into Calcite via an Amorphous Calcium Carbonate Crystallization Pathway. Cryst. Growth Des. 2017, 17 (3), 1214–1223. 10.1021/acs.cgd.6b01599.

[ref44] Campana S. E. Chemistry and composition of fish otoliths: Pathways, mechanisms and applications. Mar. Ecol.: Prog. Ser. 1999, 188, 263–297. 10.3354/meps188263.

[ref45] Greegor R. B.; Pingitore N. E.; Lytle F. W. Strontianite in Coral Skeletal Aragonite. Science (80-). 1997, 275 (5305), 1452–1454. 10.1126/science.275.5305.1452. 9072808

[ref46] Kinsman D. J. J. Interpretation of Sr+2 Concentrations in Carbonate Minerals and Rocks. J. Sediment Petrol. 1969, 39 (2), 468–508. 10.1306/74D71CB7-2B21-11D7-8648000102C1865D.

[ref47] Brinza L.; Quinn P. D.; Schofield P. F.; Mosselmans J. F. W.; Hodson M. E. Incorporation of strontium in earthworm-secreted calcium carbonate granules produced in strontium-amended and strontium-bearing soil. Geochim. Cosmochim. Acta 2013, 113, 21–37. 10.1016/j.gca.2013.03.011.

[ref48] Sunagawa I.; Takahashi Y.; Imai H. Strontium and aragonite-calcite precipitation. J. Mineral Petrol Sci. 2007, 102 (3), 174–181. 10.2465/jmps.060327a.

[ref49] Finch A. A.; Allison N.; Sutton S. R.; Newville M. Strontium in coral aragonite: 1. Characterization of Sr coordination by extended absorption X-ray fine structure. Geochim. Cosmochim. Acta 2003, 67 (6), 1197–1202. 10.1016/S0016-7037(02)01224-3.

[ref50] Foster L. C.; Allison N.; Finch A. A.; Andersson C. Strontium distribution in the shell of the aragonite bivalve *Arctica islandica*. Geochemistry, Geophys Geosystems. 2009, 10 (3), 1 10.1029/2007GC001915.

[ref51] Finch A. A.; Shaw P. A.; Holmgren K.; Lee-Thorp J. Corroborated rainfall records from aragonitic stalagmites. Earth Planet Sci. Lett. 2003, 215 (1–2), 265–273. 10.1016/S0012-821X(03)00431-X.

[ref52] Hodkin D. J.; Stewart D. I.; Graham J. T.; Cibin G.; Burke I. T. Enhanced Crystallographic Incorporation of Strontium(II) Ions into Calcite via Preferential Adsorption at Obtuse Growth Steps. Cryst. Growth Des. 2018, 18 (5), 2836–2843. 10.1021/acs.cgd.7b01614.

[ref53] Marxen J. C.; Becker W.; Finke D.; Hasse B.; Epple M. Microscopic and Structural Results. J. Moll Stud. 2003, 69, 113–121. 10.1093/mollus/69.2.113.

[ref54] Hasse B.; Ehrenberg H.; Marxen J. C.; Becker W.; Epple M. Calcium carbonate modifications in the mineralized shell of the freshwater snail *Biomphalaria glabrata*. Chem.—Eur. J. 2000, 6 (20), 3679–3685. 10.1002/1521-3765(20001016)6:20<3679::AID-CHEM3679>3.0.CO;2-#. 11073237

[ref55] Becker A.; Bismayer U.; Epple M.; et al. Structural characterisation of X-ray amorphous calcium carbonate (ACC) in sternal deposits of the crustacea *Porcellio scaber*. J. Chem. Soc. Dalt Trans. 2003, 3 (4), 551–555. 10.1039/b210529b.

[ref56] Ferris F. G.; Fratton C. M.; Gerits J. P.; Schultze-Lam S.; Lollar B. S. Microbial precipitation of a strontium calcite phase at a groundwater discharge zone near rock Creek, British Columbia, Canada. Geomicrobiol J. 1995, 13 (1), 57–67. 10.1080/01490459509378004.

[ref57] Ferris F. G.; Phoenix V.; Fujita Y.; Smith R. W. Kinetics of calcite precipitation induced by ureolytic bacteria at 10 to 20°C in artificial groundwater. Geochim. Cosmochim. Acta 2004, 68 (8), 1701–1710. 10.1016/S0016-7037(03)00503-9.

[ref58] Warren L. A.; Maurice P. A.; Parmar N.; Ferris F. G. Microbially mediated calcium carbonate precipitation: Implications for Interpreting calcite precipitation and for solid-phase capture of inorganic contaminants. Geomicrobiol J. 2001, 18 (1), 93–115. 10.1080/01490450151079833.

[ref59] Tobler D. J.; Cuthbert M. O.; Greswell R. B.; et al. Comparison of rates of ureolysis between *Sporosarcina pasteurii* and an indigenous groundwater community under conditions required to precipitate large volumes of calcite. Geochim. Cosmochim. Acta 2011, 75 (11), 3290–3301. 10.1016/j.gca.2011.03.023.

[ref60] De Muynck W.; De Belie N.; Verstraete W. Microbial carbonate precipitation in construction materials: A review. Ecol Eng. 2010, 36 (2), 118–136. 10.1016/j.ecoleng.2009.02.006.

[ref61] Zhong L.; Islam M. R. A New Microbial Plugging Process and Its Impact on Fracture Remediation. In Paper presented at the SPE Annual Technical Conference and Exhibition, Dallas, Texas, October 1995; pp 703–715; 10.2523/30519-MS.

[ref62] Raveh-Amit H.; Tsesarsky M. Biostimulation in desert soils for microbial-induced calcite precipitation. Appl. Sci. 2020, 10 (8), 2905 10.3390/app10082905.

[ref63] Atlas R. M.; Bartha R. Stimulated Biodegradation of Oil Slicks Using Oleophilic Fertilizers. Environ. Sci. Technol. 1973, 7 (6), 538–541. 10.1021/es60078a005. 22217285

[ref64] Fujita Y.; Taylor J. L.; Gresham T. L. T.; et al. Stimulation of microbial urea hydrolysis in groundwater to enhance calcite precipitation. Environ. Sci. Technol. 2008, 42 (8), 3025–3032. 10.1021/es702643g. 18497161

[ref65] Fujita Y.; Taylor J. L.; Wendt L. M.; Reed D. W.; Smith R. W. Evaluating the potential of native ureolytic microbes to remediate a 90Sr contaminated environment. Environ. Sci. Technol. 2010, 44 (19), 7652–7658. 10.1021/es101752p. 20815389

[ref66] Mitchell A. C.; Ferris F. G. The coprecipitation of Sr into calcite precipitates induced by bacterial ureolysis in artificial groundwater: temperature and kinetic dependence. Geochim. Cosmochim. Acta 2005, 69 (17), 4199–4210. 10.1016/j.gca.2005.03.014.

[ref67] Fujita Y.; Redden G.; Ingram J.; Cortez M.; Ferris G.; Smith R. Strontium incorporation into calcite generated by bacterial ureolysis. Geochim. Cosmochim. Acta 2004, 68 (15), 3261–3270. 10.1016/j.gca.2003.12.018.

[ref68] Smith N. T.; Shreeve J.; Kuras O. Multi-sensor core logging (MSCL) and X-ray computed tomography imaging of borehole core to aid 3D geological modelling of poorly exposed unconsolidated superficial sediments underlying complex industrial sites: An example from Sellafield nuclear site, UK. J. Appl. Geophys. 2020, 178, 104084 10.1016/j.jappgeo.2020.104084.

[ref69] Smith N. T.; Merritt J. W.; Phillips E. R. High-resolution 3D geological modelling of heterogeneity in poorly exposed glacial deposits using sedimentary and glaciotectonic architectural element analysis: a case example from Sellafield in west Cumbria, UK. Q J. Eng. Geol Hydrogeol. 2023, 56 (1), 1 10.1144/qjegh2022-022.

[ref70] Merritt J. W.; Auton C. A. An outline of the lithostratigraphy and depositional history of Quaternary deposits in the Sellafield district, west Cumbria. Proc. Yorksh Geol Soc. 2000, 53 (2), 129–154. 10.1144/pygs.53.2.129.

[ref71] Newsome L.; Morris K.; Trivedi D.; Atherton N.; Lloyd J. R. Microbial reduction of uranium(VI) in sediments of different lithologies collected from Sellafield. Appl. Geochem. 2014, 51, 55–64. 10.1016/j.apgeochem.2014.09.008.

[ref140] Robinson C.; Shaw S.; Lloyd J. R.; Graham J. G.; Morris K. M. Phosphate (Bio)mineralization Remediation of 90Sr-Contaminated Groundwaters. Environ. Sci. Technol. Water 2023, 3, 3223–3234. 10.1021/acsestwater.3c00159. PMC10580321 37854271

[ref72] Roden E. E.; Leonardo M. R.; Ferris F. G. Immobilization of strontium during iron biomineralization coupled to dissimilatory hydrous ferric oxide reduction. Geochim. Cosmochim. Acta 2002, 66 (16), 2823–2839. 10.1016/S0016-7037(02)00878-5.

[ref73] Parmar N.; Warren L. A.; Roden E. E.; Ferris F. G. Solid phase capture of strontium by the iron reducing bacteria *Shewanella alga* strain BrY. Chem. Geol. 2000, 169 (3–4), 281–288. 10.1016/S0009-2541(00)00208-4.

[ref74] Stookey L. L. Ferrozine-A New Spectrophotometric Reagent for Iron. Anal. Chem. 1970, 42 (7), 779–781. 10.1021/ac60289a016.

[ref75] Lovley D. R.; Phillips E. J. P. Rapid Assay for Microbially Reducible Ferric Iron in Aquatic Sediments. Appl. Environ. Microbiol. 1987, 53 (7), 1536–1540. 10.1128/aem.53.7.1536-1540.1987. 16347384 PMC203906

[ref76] Knorst M. T.; Neubert R.; Wohlrab W. Analytical methods for measuring urea in pharmaceutical formulations. J. Pharm. Biomed Anal. 1997, 15 (11), 1627–1632. 10.1016/S0731-7085(96)01978-4. 9260657

[ref77] Ravel B.; Newville M. ATHENA, ARTEMIS, HEPHAESTUS: Data analysis for X-ray absorption spectroscopy using IFEFFIT. J. Synchrotron Radiat. 2005, 12 (4), 537–541. 10.1107/S0909049505012719. 15968136

[ref78] Downward L.; Booth C. H.; Lukens W. W.; Bridges F. A variation of the F-test for determining statistical relevance of particular parameters in EXAFS fits. AIP Conf Proc. 2007, 882, 129–131. 10.1063/1.2644450.

[ref79] Parkhurst D. L.; Appelo C. A. J. User’s Guide to PHREEQC (Version 2): A Computer Program for Speciation, Batch-Reaction, One-Dimensional, Transport, and Inverse Geochemical Calculations; Water-Resources Investigations Report 99-4259; USGS, 1999.

[ref80] Giffaut E.; Grivé M.; Blanc P.; et al. Andra thermodynamic database for performance assessment: ThermoChimie. Appl. Geochem. 2014, 49 (May), 225–236. 10.1016/j.apgeochem.2014.05.007.

[ref81] Byrd N.; Lloyd J. R.; Small J. S.; et al. Microbial Degradation of Citric Acid in Low Level Radioactive Waste Disposal: Impact on Biomineralization Reactions. Front Microbiol. 2021, 12 (April), 1–14. 10.3389/fmicb.2021.565855. PMC8114274 33995289

[ref82] Cleary A.; Newsome L.; Shaw S.; et al. Bioremediation of strontium and technetium contaminated groundwater using glycerol phosphate. Chem. Geol. 2019, 509, 213–222. 10.1016/j.chemgeo.2019.02.004.

[ref83] McCarty G. W.; Bremner J. M. Production of urease by microbial activity in soils under aerobic and anaerobic conditions. Biol. Fertil Soils. 1991, 11 (3), 228–230. 10.1007/BF00335772.

[ref84] Lovley D. R. Organic matter mineralization with the reduction of ferric iron. Geomicrobiol J. 1987, 5 (3-4), 375–399. 10.1080/01490458709385975. PMC238947 16347032

[ref85] Chapatwala K. D.; Babu GR V.; Vijaya O. K.; et al. Effect of temperature and yeast extract on microbial respiration of sediments from a shallow coastal subsurface and vadose zone. Seventeenth Symposium on Biotechnology for Fuels and Chemicals 1996, 827–835. 10.1007/978-1-4612-0223-3_76. 8669920

[ref86] Gomez M. G.; Graddy C. M. R.; DeJong J. T.; Nelson D. C.; Tsesarsky M. Stimulation of Native Microorganisms for Biocementation in Samples Recovered from Field-Scale Treatment Depths. J. Geotech Geoenvironmental Eng. 2018, 144 (1), 1–13. 10.1061/(ASCE)GT.1943-5606.0001804.

[ref87] Horiike T.; Dotsuta Y.; Nakano Y.; et al. Removal of soluble strontium via incorporation into biogenic carbonate minerals by halophilic bacterium *Bacillus* sp. strain TK2d in a highly saline solution. Appl. Environ. Microbiol. 2017, 83 (20), 1–11. 10.1128/AEM.00855-17. PMC5627000 28802269

[ref88] Lauchnor E. G.; Schultz L. N.; Bugni S.; Mitchell A. C.; Cunningham A. B.; Gerlach R. Bacterially induced calcium carbonate precipitation and strontium coprecipitation in a porous media flow system. Environ. Sci. Technol. 2013, 47 (3), 1557–1564. 10.1021/es304240y. 23282003

[ref89] Su Z.; Deng Z.; Wang Y.; et al. Effects of the Sr/Ca ratio on the bioremediation of strontium based on microbially-induced carbonate precipitation. J. Environ. Chem. Eng. 2023, 11 (1), 108990 10.1016/j.jece.2022.108990.

[ref90] Fujita Y.; Ferris F.G.; Lawson R.D.; Colwell F.S.; Smith R.W. Subscribed content calcium carbonate precipitation by ureolytic subsurface bacteria. Geomicrobiol J. 2000, 17 (4), 305–318. 10.1080/782198884.

[ref91] Mitchell A. C.; Espinosa-Ortiz E. J.; Parks S. L.; Phillips A. J.; Cunningham A. B.; Gerlach R. Kinetics of calcite precipitation by ureolytic bacteria under aerobic and anaerobic conditions. Biogeosciences. 2019, 16 (10), 2147–2161. 10.5194/bg-16-2147-2019.

[ref92] Gal J. Y.; Bollinger J. C.; Tolosa H.; Gache N. Calcium carbonate solubility: A reappraisal of scale formation and inhibition. Talanta. 1996, 43 (9), 1497–1509. 10.1016/0039-9140(96)01925-X. 18966629

[ref93] Coto B.; Martos C.; Peña J. L.; Rodríguez R.; Pastor G. Effects in the solubility of CaCO 3: Experimental study and model description. Fluid Phase Equilib. 2012, 324, 1–7. 10.1016/j.fluid.2012.03.020.

[ref94] Blue C. R.; Giuffre A.; Mergelsberg S.; Han N.; De Yoreo J. J.; Dove P. M. Chemical and physical controls on the transformation of amorphous calcium carbonate into crystalline CaCO3 polymorphs. Geochim. Cosmochim. Acta 2017, 196, 179–196. 10.1016/j.gca.2016.09.004.

[ref95] Mitchell A. C.; Ferris F. G. Effect of strontium contaminants upon the size and solubility of calcite crystals precipitated by the bacterial hydrolysis of urea. Environ. Sci. Technol. 2006, 40 (3), 1008–1014. 10.1021/es050929p. 16509350

[ref96] Mitchell A. C.; Ferris G. F. The coprecipitation of Sr into calcite precipitates induced by bacterial ureolysis in artificial groundwater: Temperature and kinetic dependence. Geochim. Cosmochim. Acta 2005, 69 (17), 4199–4210. 10.1016/j.gca.2005.03.014.

[ref97] Sheng Han Y.; Hadiko G.; Fuji M.; Takahashi M. Crystallization and transformation of vaterite at controlled pH. J. Cryst. Growth. 2006, 289 (1), 269–274. 10.1016/j.jcrysgro.2005.11.011.

[ref98] Xyla A. G.; Koutsoukos P. G. Quantitative analysis of calcium carbonate polymorphs by infrared spectroscopy. J. Chem. Soc. Faraday Trans 1 Phys. Chem. Condens Phases. 1989, 85 (10), 3165–3172. 10.1039/f19898503165.

[ref99] Sondi I.; Salopek-Sondi B. Influence of the primary structure of enzymes on the formation of CaCO 3 polymorphs: A comparison of plant (*Canavalia ensiformis*) and bacterial (*Bacillus pasteurii*) ureases. Langmuir. 2005, 21 (19), 8876–8882. 10.1021/la051129v. 16142973

[ref100] Sondi I.; Matijević E. Homogeneous precipitation of calcium carbonates by enzyme catalyzed reaction. J. Colloid Interface Sci. 2001, 238 (1), 208–214. 10.1006/jcis.2001.7516. 11350156

[ref101] Rodriguez-Blanco J. D.; Shaw S.; Bots P.; Roncal-Herrero T.; Benning L. G. The role of pH and Mg on the stability and crystallization of amorphous calcium carbonate. J. Alloys Compd. 2012, 536 (SUPPL.1), S477–S479. 10.1016/j.jallcom.2011.11.057.

[ref102] Demichelis R.; Raiteri P.; Gale J.; Dovesi R. A new structural model for disorder in vaterite from first-principles calculations. CrystEngComm. 2012, 14 (1), 44–47. 10.1039/C1CE05976A.

[ref103] Shuseki Y.; Kohara S.; Ohara K.; et al. Structural analyses of amorphous calcium carbonate before and after removing strontium ions from an aqueous solution. J. Ceram Soc. Japan. 2022, 130 (2), 225–231. 10.2109/jcersj2.21155.

[ref104] Wallace S. H.; Shaw S.; Morris K.; Small J. S.; Fuller A. J.; Burke I. T. Effect of groundwater pH and ionic strength on strontium sorption in aquifer sediments: Implications for 90Sr mobility at contaminated nuclear sites. Appl. Geochem. 2012, 27 (8), 1482–1491. 10.1016/j.apgeochem.2012.04.007.

[ref105] Fuller A. J.; Shaw S.; Peacock C. L.; Trivedi D.; Burke I. T. EXAFS Study of Sr sorption to Illite, Goethite, Chlorite, and Mixed Sediment under Hyperalkaline Conditions. Langmuir. 2016, 32 (12), 2937–2946. 10.1021/acs.langmuir.5b04633. 26938867

[ref106] Blanco-Gutierrez V.; Demourgues A.; Jubera V.; Gaudon M. Eu(III)/Eu(II)-doped (Ca0.7Sr0.3)CO3 phosphors with vaterite/calcite/aragonite forms as shock/temperature detectors. J. Mater. Chem. C 2014, 2 (46), 9969–9977. 10.1039/C4TC01382D.

[ref107] Mougoyannis M. M.; Kroner A.; Dowding P. J. Structure of Non-Crystalline CaCO3 Products by X-ray Absorption Spectroscopy. In MSNGS-Methods to simulate nucleation and growth from solutions, Sheffield, UK, 2016.

[ref108] Zhang Y.; Qiao L.; Yan H.; et al. Vaterite Microdisc Mesocrystals Exposing the (001) Facet Formed via Transformation from Proto-Vaterite Amorphous Calcium Carbonate. Cryst. Growth Des. 2020, 20 (5), 3482–3492. 10.1021/acs.cgd.0c00259.

[ref109] Gebauer D.; Gunawidjaja P. N.; Ko J. Y. P.; et al. Proto-calcite and proto-vaterite in amorphous calcium carbonates. Angew. Chemie - Int. Ed. 2010, 49 (47), 8889–8891. 10.1002/anie.201003220. 20949576

[ref110] Qiao L.; Zizak I.; Zaslansky P.; Ma Y. The crystallization process of vaterite microdisc mesocrystals via proto-vaterite amorphous calcium carbonate characterized by cryo-x-ray absorption spectroscopy. Crystals. 2020, 10 (9), 750 10.3390/cryst10090750.

[ref111] Kamhi S. R. On the structure of vaterite CaCO 3. Acta Crystallogr. 1963, 16 (8), 770–772. 10.1107/S0365110X63002000.

[ref112] Lam R. S. K.; Charnock J. M.; Lennie A.; Meldrum C. Synthesis-dependant structural variations in amorphous calcium carbonate. CrystEngComm. 2007, 9 (12), 1226–1236. 10.1039/b710895h.

[ref113] Achal V.; Pan X.; Zhang D. Bioremediation of strontium (Sr) contaminated aquifer quartz sand based on carbonate precipitation induced by Sr resistant *Halomonas* sp. Chemosphere. 2012, 89 (6), 764–768. 10.1016/j.chemosphere.2012.06.064. 22850277

[ref114] Kang C. H.; Choi J. H.; Noh J. G.; Kwak D. Y.; Han S. H.; So J. S. Microbially Induced Calcite Precipitation-based Sequestration of Strontium by *Sporosarcina pasteurii* WJ-2. Appl. Biochem. Biotechnol. 2014, 174 (7), 2482–2491. 10.1007/s12010-014-1196-4. 25190302

[ref115] Dalas E.; Koutsoukos P. G. Calcium Carbonate Scale Formation on Heated Metal Surfaces. Geothermics. 1989, 18 (1–2), 83–88. 10.1016/0375-6505(89)90013-8.

[ref116] Zhou G.-T.; Yao Q.-Z.; Fu S.-Q.; Guan Y.-B. Controlled crystallization of unstable vaterite with distinct morphologies and their polymorphic transition to stable calcite. Eur. J. Mineral. 2010, 22 (2), 259–269. 10.1127/0935-1221/2009/0022-2008.

[ref117] Matsunuma S.; Kagi H.; Komatsu K.; Maruyama K.; Yoshino T. Doping incompatible elements into calcite through amorphous calcium carbonate. Cryst. Growth Des. 2014, 14 (11), 5344–5348. 10.1021/cg500953h.

[ref118] Finch A. A.; Allison N. Coordination of Sr and Mg in calcite and aragonite. Mineral Mag. 2007, 71 (5), 539–552. 10.1180/minmag.2007.071.5.539.

[ref119] Thorpe C. L.; Lloyd J. R.; Law G. T. W.; et al. Strontium sorption and precipitation behaviour during bioreduction in nitrate impacted sediments. Chem. Geol. 2012, 306, 114–122. 10.1016/j.chemgeo.2012.03.001.

[ref120] Khatibikamal V.; Panahi H. A.; Torabian A.; Baghdadi M. Optimized poly(amidoamine) coated magnetic nanoparticles as adsorbent for the removal of nonylphenol from water. Microchem J. 2019, 145, 508–516. 10.1016/j.microc.2018.11.018.

[ref121] Hata T.; Saracho A. C.; Haigh S. K.; Yoneda J.; Yamamoto K. Microbial-induced carbonate precipitation applicability with the methane hydrate-bearing layer microbe. J. Nat. Gas Sci. Eng. 2020, 81 (July), 103490 10.1016/j.jngse.2020.103490.

[ref122] Cuaxinque-Flores G.; Aguirre-Noyola J. L.; Hernández-Flores G.; Martínez-Romero E.; Romero-Ramírez Y.; Talavera-Mendoza O. Bioimmobilization of toxic metals by precipitation of carbonates using *Sporosarcina luteola*: An in vitro study and application to sulfide-bearing tailings. Sci. Total Environ. 2020, 724, 138124 10.1016/j.scitotenv.2020.138124. 32268286

[ref123] Castro-Alonso M. J.; Montañez-Hernandez L. E.; Sanchez-Muñoz M. A.; Macias Franco M. R.; Narayanasamy R.; Balagurusamy N. Microbially induced calcium carbonate precipitation (MICP) and its potential in bioconcrete: Microbiological and molecular concepts. Front Mater. 2019, 6 (June), 1–15. 10.3389/fmats.2019.00126.

[ref124] Omoregie A. Bacteria Isolated from Limestone Caves of Sarawak and Evaluation of Their Efficiency. MSc. Thesis, 2016; 10.13140/RG.2.2.11719.55205.

[ref125] Skorupa D. J.; Akyel A.; Fields M. W.; Gerlach R. Facultative and anaerobic consortia of haloalkaliphilic ureolytic micro-organisms capable of precipitating calcium carbonate. J. Appl. Microbiol. 2019, 127 (5), 1479–1489. 10.1111/jam.14384. 31301204

[ref126] Ghosh T.; Bhaduri S.; Montemagno C.; Kumar A. *Sporosarcina pasteurii* can form nanoscale calcium carbonate crystals on cell surface. PLoS One. 2019, 14 (1), e0210339 10.1371/journal.pone.0210339. 30699142 PMC6353136

[ref127] Achal V.; Mukherjee A.; Basu P. C.; Reddy M. S. Strain improvement of *Sporosarcina pasteurii* for enhanced urease and calcite production. J. Ind. Microbiol Biotechnol. 2009, 36 (7), 981–988. 10.1007/s10295-009-0578-z. 19408027

[ref128] Gat D.; Ronen Z.; Tsesarsky M. Soil Bacteria Population Dynamics Following Stimulation for Ureolytic Microbial-Induced CaCO3 Precipitation. Environ. Sci. Technol. 2016, 50 (2), 616–624. 10.1021/acs.est.5b04033. 26689904

[ref129] Greenwood J. A.; Mills J.; Tyler P. D.; Jones C. W. Physiological regulation, purification and properties of urease from *Methylophilus methylotrophus*. FEMS Microbiol Lett. 1998, 160 (1), 131–135. 10.1016/S0378-1097(98)00022-6.

[ref130] Keykha H. A.; Huat B. B. K.; Asadi A.; Zareian M.; Kawasaki S. Electrokinetic properties of pasteurii and aquimarina bacteria. Environ. Geotech. 2015, 2 (3), 181–188. 10.1680/envgeo.13.00072.

[ref131] Graddy C. M. R.; Gomez M. G.; Kline L. M.; Morrill S. R.; Dejong J. T.; Nelson D. C. Diversity of *Sporosarcina* -like Bacterial Strains Obtained from Meter-Scale Augmented and Stimulated Biocementation Experiments. Environ. Sci. Technol. 2018, 52 (7), 3997–4005. 10.1021/acs.est.7b04271. 29505251

[ref132] Achal V.; Pan X.; Fu Q.; Zhang D. Biomineralization based remediation of As(III) contaminated soil by *Sporosarcina ginsengisoli*. J. Hazard Mater. 2012, 201–202, 178–184. 10.1016/j.jhazmat.2011.11.067. 22154871

[ref133] Wang S.; Zhu G.; Zhuang L.; et al. Anaerobic ammonium oxidation is a major N-sink in aquifer systems around the world. ISME J. 2020, 14 (1), 151–163. 10.1038/s41396-019-0513-x. 31595050 PMC6908648

[ref134] Sawayama S. Possibility of anoxic ferric ammonium oxidation. J. Biosci Bioeng. 2006, 101 (1), 70–72. 10.1263/jbb.101.70. 16503294

[ref135] Yang W. H.; Weber K. A.; Silver W. L. Nitrogen loss from soil through anaerobic ammonium oxidation coupled to iron reduction. Nat. Geosci. 2012, 5 (8), 538–541. 10.1038/ngeo1530.

[ref136] Newsome L.; Morris K.; Trivedi D.; Bewsher A.; Lloyd J. Biostimulation by glycerol phosphate to precipitate recalcitrant uranium (IV) phosphate. Environ. Sci. Technol. 2015, 49 (18), 11070–11078. 10.1021/acs.est.5b02042. 26292021

[ref137] Latta D. E.; Boyanov M. I.; Kemner K. M.; O’Loughlin E. J.; Scherer M. M. Abiotic reduction of uranium by Fe(II) in soil. Appl. Geochem. 2012, 27 (8), 1512–1524. 10.1016/j.apgeochem.2012.03.003.

[ref138] McBeth J. M.; Lear G.; Lloyd J. R.; Livens F. R.; Morris K.; Burke I. T. Technetium reduction and reoxidation in aquifer sediments. Geomicrobiol J. 2007, 24 (3–4), 189–197. 10.1080/01490450701457030.

[ref139] Brookshaw D. R.; Pattrick R. A. D.; Bots P.; et al. Redox Interactions of Tc(VII), U(VI), and Np(V) with Microbially Reduced Biotite and Chlorite. Environ. Sci. Technol. 2015, 49 (22), 13139–13148. 10.1021/acs.est.5b03463. 26488884

