# Supplementary material for: Enhanced Strontium Removal through Microbially Induced Carbonate Precipitation by Indigenous Ureolytic Bacteria
Source: ACS Earth Space Chem. 2024 Feb 26;8(3):483–98. doi: 10.1021/acsearthspacechem.3c00252 (PMC10961847; doi:10.1021/acsearthspacechem.3c00252)
Supplement: Supplementary file 1 — sp3c00252_si_001.pdf [file sp3c00252_si_001.pdf]

Supporting information for

Enhanced strontium removal through microbially induced carbonate precipitation by indigenous ureolytic bacteria

Matthew White-Pettigrew[1], Samuel Shaw[1], Lewis Hughes[1], Christopher Boothman[1†], James Graham[2], Liam Abrahamsen-Mills[2], Katherine Morris[1], Jonathan R. Lloyd[1]*

1 Research Centre for Radwaste Disposal and Williamson Research Centre for Molecular Environmental Science, Department of Earth and Environmental Sciences, The University of Manchester, Manchester, M13 9PL, U.K.

2 National Nuclear Laboratory, Warrington, Cheshire, WA3 6AE, U.K.

Corresponding author:
* Jon.lloyd@manchester.ac.uk

Microbial ecology methods

A PowerSoil DNA isolation kit (MO Bio, USA) was used to perform DNA extractions, allowing for the amplification of the 16s rRNA gene using the universal primers 8F and 1429R [1]. Products amplified during the polymerase chain reaction (PCR) were separated and analysed for their purity using electrophoresis in Tris-acetate-EDTA gel. Quantification of the amplified PCR products and sequencing of the 16S rRNA genes were conducted using the Illumina MiSeq platform (Illumina, San Diego, CA, USA), which targeted the V4 hyper variable region (forward primer, 515F, 5′-GTGYCAGCMGCCGCGGTAA-3′; reverse primer, 806R, 5′-GGACTACHVGGGTWTCTAAT-3′) for 2 × 250-bp paired-end sequencing (Illumina) [2,3].

PCR amplification was performed using Roche FastStart High Fidelity PCR System (Roche Diagnostics Ltd, Burgess Hill, UK) in 50µl reactions under the following conditions; initial denaturation at 95°C for 2 min, followed by 36 cycles of 95°C for 30 s, 55°C for 30 s, 72°C for 1 min, and a final extension step of 5 min at 72°C. The PCR products were purified and normalised to ~20ng each using the SequalPrep Normalization Kit (Fisher Scientific, Loughborough, UK). The PCR amplicons from all samples were pooled in equimolar ratios. The run was performed using a 4pM sample library spiked with 4pM PhiX to a final concentration of 10% [4].

Raw sequences were divided into samples by barcodes (up to one mismatch was permitted) using a sequencing pipeline. Quality control and trimming was performed using Cutadapt [5], FastQC (https://www.bioinformatics.babraham.ac.uk/projects/fastqc/), and Sickle [6]. MiSeq error correction was performed using SPADes [7]. Forward and reverse reads were incorporated into full-length sequences with Pandaseq [8]. Chimeras were removed using ChimeraSlayer [9], and OTUs were generated with UPARSE [10]. OTUs were classified by Usearch [11] at the 97% similarity level, and singletons were removed. Rarefaction analysis was conducted using the original detected OTUs in Qiime [12]. The taxonomic assignment was performed by the RDP classifier [13] and the closest GenBank matches identified by Blastn nucleotide search (http://blast.ncbi.nlm.nih.gov). Sequences were compared to known phylogeny lists of microbial metabolic function [14–17]. The method follows that of Cleary *et al.,* [18].

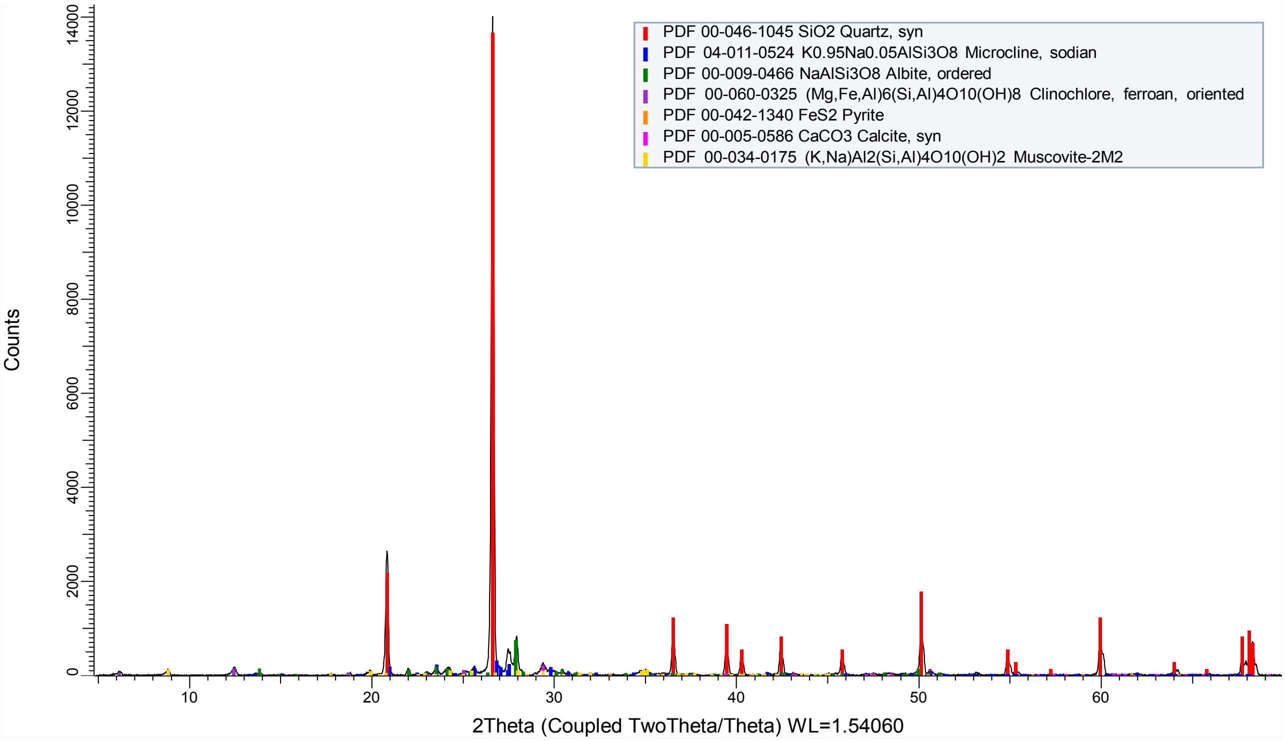

Figure S1. XRD pattern of the sediments obtained from the Peel Place Quarry.

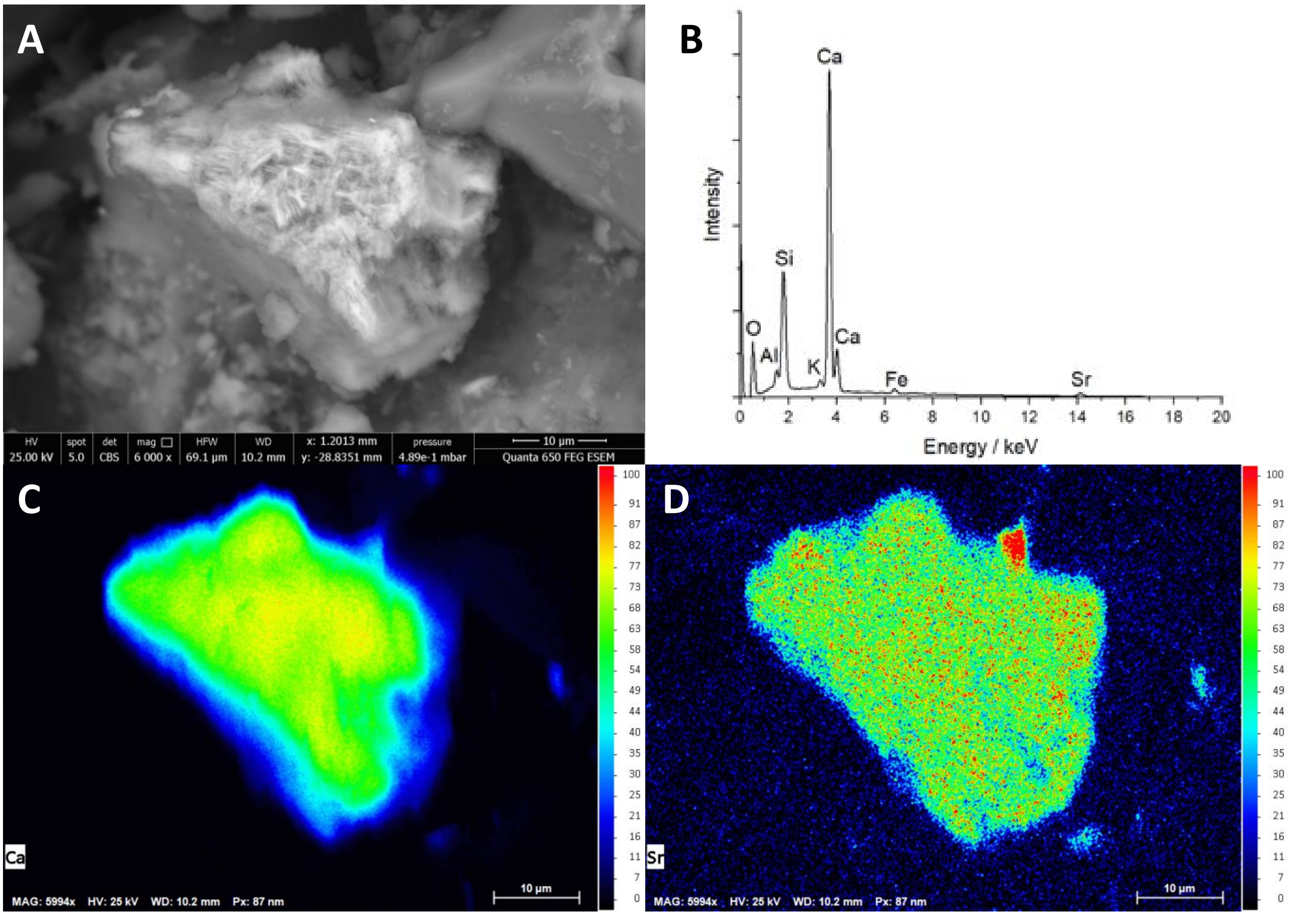

Figure S2. ESEM analysis of a rhombohedral precipitate formed in System E for RB27 sediments, 17 days after incubation. (a) Image in backscatter mode. (b) The corresponding EDX spectrum and (c and d) elemental mapping revealing a close correlation between Ca and Sr.

```
Database Thermochimie.dat

SOLUTION_MASTER_SPECIES # Urea added to the solution master species block.
    Urea        Urea    0.0     NH2CONH2        60.06

SOLUTION_SPECIES # Identify reaction to ensure urea is recognised as an aqueous species.

Urea = Urea
        log_k   0.0


RATES


# d[Urea]/dt = -k_U * [Urea], where k_U = bacterial ureolysis rate constant = 0.04 per day (Tobler et al., 2011)

  U_degradation
 -start
 10 k_U = 4.6e-7 # units = per second
 20 Ur = tot("Urea")
 30 rate = - k_U * Ur s
 40 moles = rate * time
 50 save moles
 -end

SOLUTION 1

# Composition of Sellafield groundwater spiked with 1 mM strontium and excess urea

-temp      10
-pH        6.5
-pe        4
-redox     pe
-units     mmol/kgw
-density   1
-water     1 # kg

   Ca         0.68
   Mg         0.23
   Na         1.49
   C(+4)      0.96
   Cl         1.48
   N(+5)      0.34
   S(+6)      0.26
   K          0.13
   Urea       20
   Sr         1.14



PHASES
        Strontianite
                SrCO3 = CO3-2 + Sr+2
                log_k           -9.271

KINETICS 1

U_degradation

-formula Urea 1 H2O 2 NH3 -2 # NH2CONH2 + 2H2O = 2NH4+ + CO3-2
-steps 85000 in 50 # steps = 1 day in seconds

INCREMENTAL_REACTIONS true


SELECTED_OUTPUT
    -file                 sr_ureolysis_nh3_pgo.xls
    -reset                false
    -pH                   true
    -alkalinity           true
    -step                 true
    -time                 true
    -saturation_indices   Calcite Aragonite Strontianite Vaterite
    -molalities  Ca+2 Sr+2 NH4+  NH3  CO3-2 HCO3-
    -totals N(-3) Urea  C

END
```

Figure S3. PHREEQC input file for the microbial hydrolysis of urea in Sellafield groundwater containing 1 mM strontium [19]. A ureolysis rate constant of 0.04 per day was used in this simulation, derived from analysing environmental samples [20].

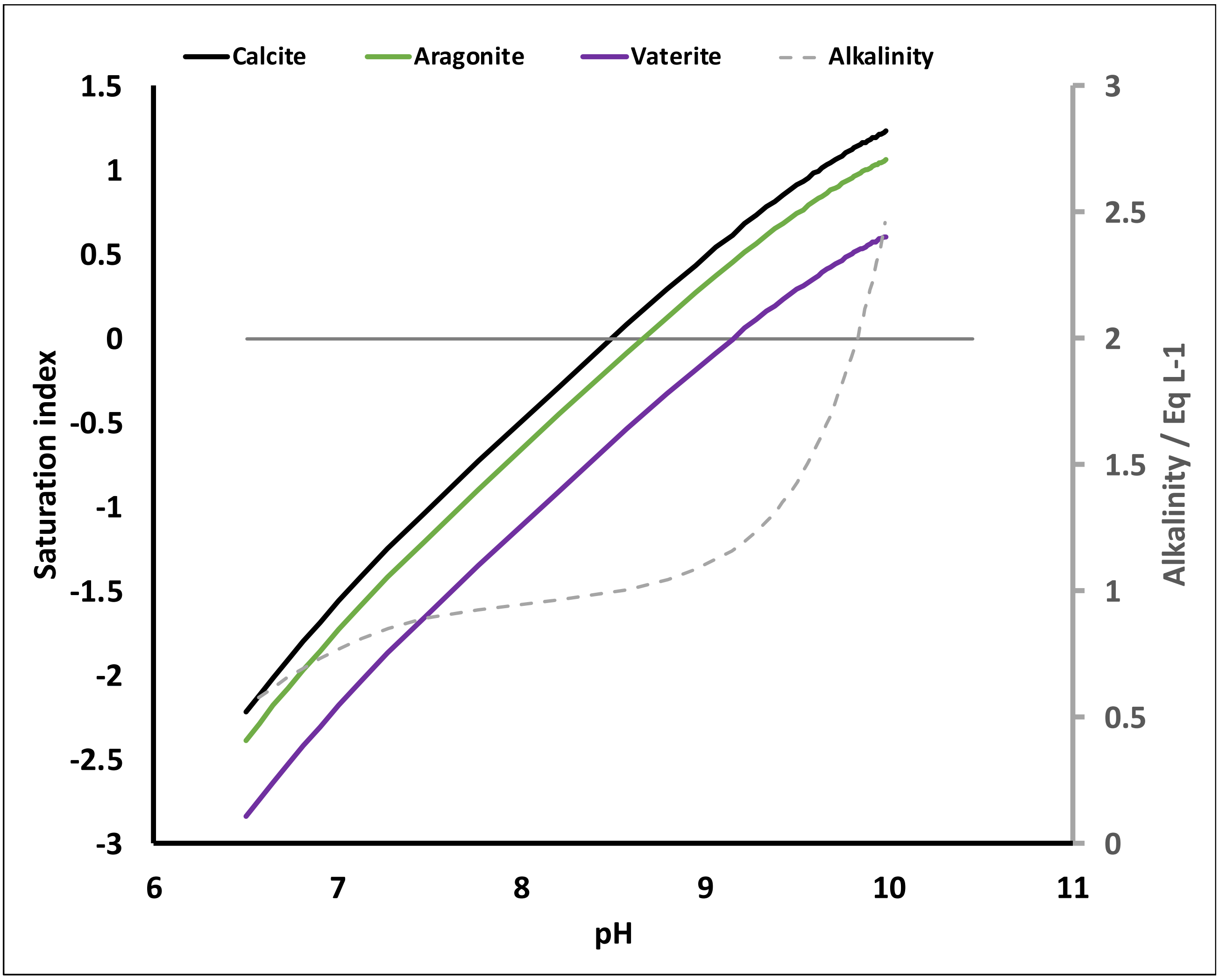

Figure S4. Evolution of alkalinity and saturation indices for relevant carbonate minerals during ureolysis in Sellafield groundwater, modelled using PHREEQC [19].

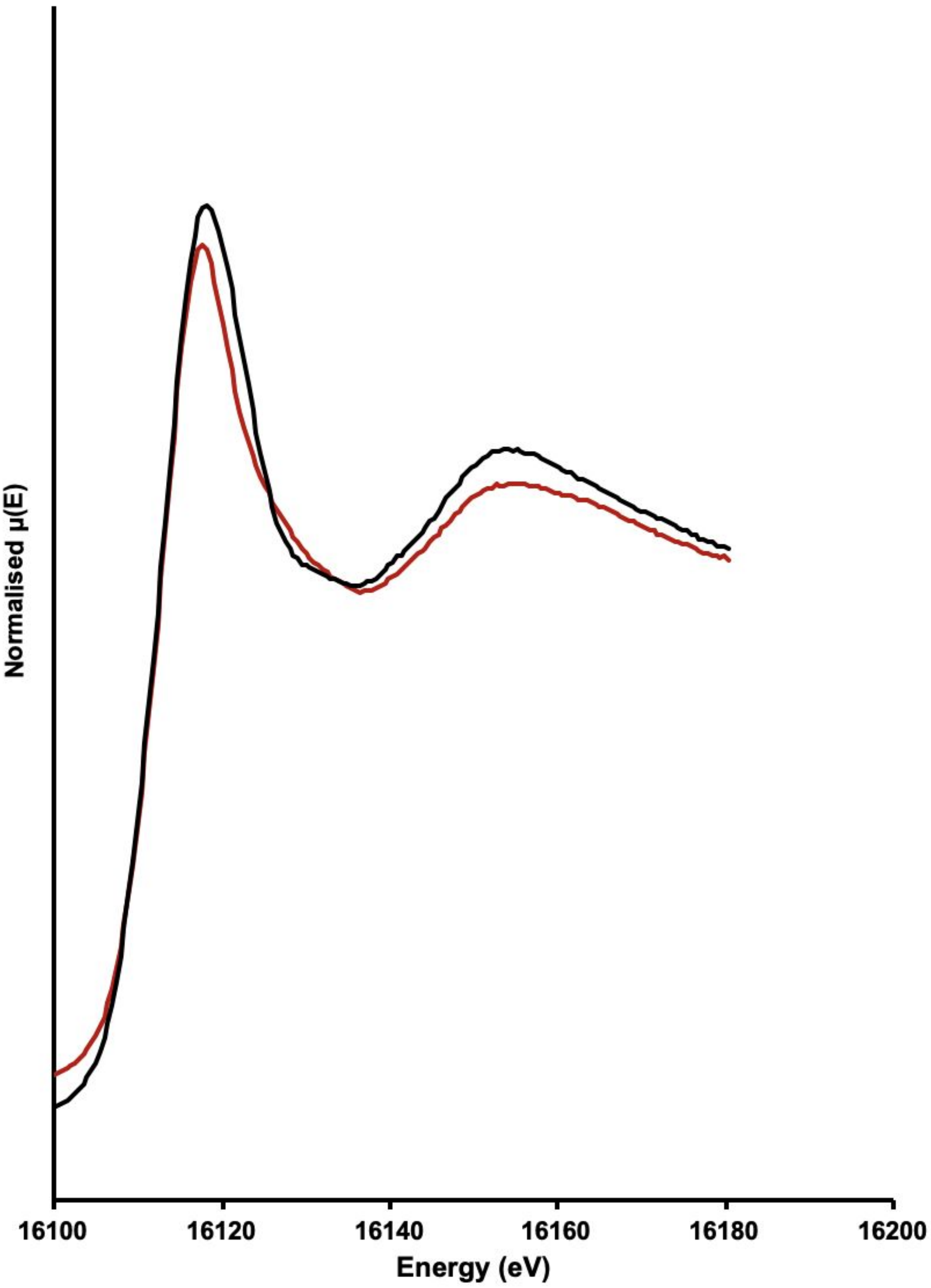

Figure S5. Normalised Sr *K*-edge XANES for biostimulated sediments extracted from the RB27 system amended with 10 mM Fe(III) (red) and the PPQ system amended with 1 g/L yeast extract (black), 17 days after incubation.

Table S1. Further details of EXAFS Fits obtained for Sr-bearing sediments in the respective PPQ and RB27 systems.[a]

**-Sediment / system**<br>**-Fit description**	**Coordination path**	**Coordination number**	**R(Å)**	**σ^2(Å^2)**	**ΔE_0(eV)**	**R-factor ("goodness of fit")**	**confidence level of adding shell (α)[b]**
**PPQ**<br>**Sr sorption standard**	Sr-O	9	2.60	0.012	-3.386	0.016	-
**PPQ**<br>**Sr-incorporated vaterite***	Sr-O	8	2.53	0.010	6.286	0.105	-
	Sr-C	4	2.95	0.006	6.286	0.054	1.00
	Sr-Ca	2 (4)	3.95 (4.03)	0.007 (0.031)	6.286	(0.040)	(0.96)
	Sr-Ca	2	4.21	0.004	6.286	0.020	1.00[*c]
**RB27 + 10 mM Fe (III)**<br>**Sr-incorporated calcite***	Sr-O	9	2.60	0.011	3.278	0.041	-
	Sr-C	5	3.03	0.030	3.278	0.032	0.91
	Sr-Ca	4	4.10	0.019	3.278	0.015	1.00

*: () = Fitting results using shell parameters in accordance with study that supported EXAFS fitting [21].

[a]: R = atomic distance, σ^2 = Debye-Waller factor, ΔE_0 = energy shift from calculated fermi level. The amplitude factor (S02) was set to 1 for each sample.

[b]: Results of the f-test: $\alpha > 0.95$ statistically improves the fit with 2σ significance, $\alpha > 0.68$ statistically improves the fit with 1σ significance.

[c]: Confidence level of splitting Sr-Ca shell (CN = 4, R = 4.03 Å, α = 0.96) into 2 separate Sr-Ca shells (CN = 2 and 2, R = 3.95 Å and 4.21 Å).

Table S2. Closest phylogenetic relatives of the five most abundant OTUs in selected PPQ systems.

OTU ID	% total population	Closest phylogenetic relative				
		**Name**	**Accession number**	**% ID similarity**	**Score**	**Description / Environment**
**Sediment only (day 0)**						
**11**	17.31%	***Nocardioides sp. SS2***	AB627754.1	100	468	Mycotoxin-degrading bacertia isolated from various soils. Genus previously exhibiting urease activity and isolated from a carbonate cave.
**4**	3.94%	***Niallia circulans strain GN03***	CP053315.1	100	468	Facultative anaerobe inhabiting plant rhizosphere.
**450**	3.75%	***Bacillus Longiquaesitum strain LMG 23783***	AM747042.1	98	446	Soil-dominant bacteria and potential methanotroph capable of forming endospores.
**86**	3.09%	***Solimonas sp. CDMK***	LT628533.1	98	446	Mn(II)-oxidising, isolated from biofilter used to remediate Mn(II)-contaminated groundwater.
**6**	2.35%	***Paenarthrobacter ureafacians strain AT63***	MZ676045.1	100	468	Bacterium isolated from As-impacted volcanic soil, producing acids that promote plant growth.
**Sediment control (day 17).**						
**17**	13.33%	***Limnobacter thiooxidans strain Na15HA-1***	KT159330.1	100	468	Species isolated from surface seawater samples.
**9**	9.69%	***Pseudorhodobacter sp. E13***	MK156742.1	100	462	Urease-producing bacterium isolated from seawater.
**13**	9.41%	***Novosphingobium aromaticivorans strain T2BR2***	JF459973.1	100	468	Species commonly associated with freshwater and seawater sediments.
**5**	8.49%	***Methylophilus methylotrophus strain 5pw***	LC191544.1	100	468	Urease-positive bacterium. Genus encoding several pathways for nitrogen acquisition from urea, ammonia and nitrate.
**6114**	6.79%	***Oxalobacteraceae bacterium AB_9***	JQ033393.1	96	412	Mn(II)-oxidising isolate from low pH former uranium mine .
**Urea only (day 17)**						
**5**	29.38%	***Methylophilus methylotrophus strain 5pw***	LC191544.1	100	468	Urease-positive bacterium. Genus encoding several pathways for nitrogen acquisition from urea, ammonia and nitrate.
**7**	18.15%	***Cavicella subterrenea strain WFH678***	MT825199.1	100	468	Novel genus isolated from hydrocarbon-contaminated soil and at depth in a mineral-water aquifer.
**18**	7.75%	***Bdellovibrio sp. Lanham***	AY094116.1	100	468	Obligate aerobe isolated from freshwater. Genus encoding a gene product that utilises urea to obtain energy.
**9**	7.71%	***Pseudorhodobacter sp. E13***	MK156742.1	100	462	Urease-producing bacterium isolated from seawater.
**25**	4.86%	***Phenylobacterium koreense strain Slu-01***	MZ369178.1	99	451	Aerobic bacterium isolated from activated sludge from a wastewater plant. Nitrate reducing bacteria capable of utilizing acetate.
**Urea + 1 g/L yeast extract (day 17)**						
**2**	14.59%	***Sporosarcina pasteurii strain NB28***	KX212192.1	100	468	Ureolytic bacterium isolated from a limestone cave.
**3**	11.84%	***Pseudomonas borbori strain R-23174***	AM114533.1	100	462	Bacterium isolated from a nitrifying inoculum.
**2117**	10.74%	***Acidovorax facilis strain IDR 3L1-3***	MT941749.1	99	451	Bacterium isolated from deep aquifer community capable of degrading nitrogenous herbicide atrazine.
**6795**	10.39%	***Sporosarcina pasteurii strain SL2_FACULANA***	MG674285.1	98	435	Ureolytic facultative anaerobe, isolated from alkaline and saline lake sediments.
**115**	8.45%	***Lentibacillus salinarum strain AHS-1***	NR_044305.1	96	418	Facultative anaerobe capable of reducing nitrate.

Table S3. Closest phylogenetic relatives of the five most abundant OTUs in selected RB27 systems.

OTU ID	% total population	Closest phylogenetic relative				
		Name	Accession number	% ID similarity	Score	Description / Environment
**Sediment only (day 0)**						
**450**	3.96%	***Bacillus Longiquaesitum strain LMG 23783***	AM747042.1	98	446	Soil-dominant bacteria and potential methanotroph capable of forming endospores.
**4**	3.70%	***Niallia circulans strain GN03***	CP053315.1	100	468	Facultative anaerobe inhabiting plant rhizosphere.
**46**	2.89%	***Panacagrimonas perspica strain Gsoil 142***	NR_112617.1	92	351	Isolated from a Korean soil, able to utilise acetate.
**6**	2.86%	***Paenarthrobacter ureafacians strain AT63***	MZ676045.1	100	468	Bacterium isolated from As-impacted volcanic soil, producing acids that promote plant growth.
**28**	2.60%	***Uncultured bacterium clone Fl-1F_H01***	EF220541.1	98	446	Isolated from slightly acidic terrestrial soil.
**Sediment control (day 17)**						
**26**	5.08%	***Sphingomonas sp. KAR7***	EF451637.1	100	468	Isolated from Arctic permafrost soil.
**35**	4.66%	***Nitrosospira multiformis strain ATCC 25196***	NR_074736.1	100	468	Soil bacterium able to oxidise ammonia
**6**	4.35%	***Paenarthrobacter ureafacians strain AT63***	MZ676045.1	100	468	Bacterium isolated from As-impacted volcanic soil, producing acids that promote plant growth.
**450**	3.71%	***Bacillus Longiquaesitum strain LMG 23783***	AM747042.1	98	446	Soil-dominant bacteria and potential methanotroph capable of forming endospores.
**3**	3.46%	***Pseudomonas borbori strain R-23174***	AM114533.1	100	462	Bacterium isolated from a nitrifying inoculum.
**Urea only (day 17)**						
**3**	10.28%	***Pseudomonas borbori strain R-23174***	AM114533.1	100	462	Bacterium isolated from a nitrifying inoculum.
**7**	6.55%	***Cavicella subterrenea strain WFH678***	MT825199.1	100	468	Novel genus isolated from hydrocarbon-contaminated soil and at depth in a mineral-water aquifer.
**450**	5.98%	***Bacillus Longiquaesitum strain LMG 23783***	AM747042.1	98	446	Soil-dominant bacteria and potential methanotroph capable of forming endospores.
**4**	5.42%	***Niallia circulans strain GN03***	CP053315.1	100	468	Facultative anaerobe inhabiting plant rhizosphere.
**6**	4.59%	***Paenarthrobacter ureafacians strain AT63***	MZ676045.1	100	468	Bacterium isolated from As-impacted volcanic soil, producing acids that promote plant growth.
**Urea + 10 mM Fe(III) (day 17)**						
**3582**	13.20%	***Sporosarcina aquimarina***	EU308120.1	98	435	Ureolytic and facultative anaerobe.
**4**	9.95%	***Niallia circulans strain GN03***	CP053315.1	100	468	Facultative anaerobe inhabiting plant rhizosphere.
**11285**	5.00%	***Sporosarcina ginsengisoli strain JCT-12***	JQ675304.1	98	440	Urease-producing bacterium able to precipitate calcite.
**2**	4.06%	***Sporosarcina pasteurii strain NB28***	KX212192.1	100	468	. Ureolytic bacterium isolated from a limestone cave.
**450**	3.63%	***Bacillus Longiquaesitum strain LMG 23783***	AM747042.1	98	446	Soil-dominant bacteria and potential methanotroph capable of forming endospores.

References

1. Lane DJ. 16S/23S rRNA sequencing. In: Stackebrant E, Goodfellow M, eds. *Nucleic Acid Techniques in Bacterial Systematics*. London: John Wiley & Sons; 1991:115–175.
2. Caporaso JG, Lauber CL, Walters WA, et al. Ultra-high-throughput microbial community analysis on the Illumina HiSeq and MiSeq platforms. *ISME J*. 2012;6(8):1621-1624. doi:10.1038/ismej.2012.8
3. Caporaso JG, Lauber CL, Walters WA, et al. Global patterns of 16S rRNA diversity at a depth of millions of sequences per sample. *Proc Natl Acad Sci U S A*. 2011;108(SUPPL. 1):4516-4522. doi:10.1073/pnas.1000080107
4. Kozich JJ, Westcott SL, Baxter NT, Highlander SK, Schloss PD. Development of a dual-index sequencing strategy and curation pipeline for analyzing amplicon sequence data on the MiSeq Illumina sequencing platform. *Appl Environ Microbiol*. 2013;79(17):5112-5120. doi:10.1128/AEM.01043-13
5. Martin M. Cutadapt removes adapter sequences from high-throughput sequencing reads. *EMBnet.journal*. 2011;17(1):10. doi:10.14806/ej.17.1.200
6. Joshi, Fass J. Sickle: A sliding-window, adaptive, quality-based trimming tool for FastQ files.
7. Nurk S, Bankevich A, Antipov D, et al. Assembling single-cell genomes and mini-metagenomes from chimeric MDA products. *J Comput Biol*. 2013;20(10):714-737. doi:10.1089/cmb.2013.0084
8. Masella AP, Bartram AK, Truszkowski JM, Brown DG, Neufeld JD. PANDAseq: paired-end assembler for Illumina sequences. *BMC Bioinformatics*. 2012;13(1):31. doi:10.1186/1471-2105-13-31
9. Haas BJ, Gevers D, Earl AM, et al. Chimeric 16S rRNA sequence formation and detection in Sanger and 454-pyrosequenced PCR amplicons. *Genome Res*. 2011;21(3):494-504. doi:10.1101/gr.112730.110
10. Edgar RC. UPARSE: highly accurate OTU sequences from microbial amplicon reads. *Nat Methods*. 2013;10(10):996-998. doi:10.1038/nmeth.2604
11. Edgar RC. Search and clustering orders of magnitude faster than BLAST. *Bioinformatics*. 2010;26(19):2460-2461. doi:10.1093/bioinformatics/btq461
12. Caporaso JG, Kuczynski J, Stombaugh J, et al. QIIME allows analysis of high-

throughput community sequencing data. *Nat Methods*. 2010;7(5):335-336. doi:10.1038/nmeth.f.303

13. Wang Q, Garrity GM, Tiedje JM, Cole JR. Naive Bayesian classifier for rapid assignment of rRNA sequences into the new bacterial taxonomy. *Appl Environ Microbiol*. 2007;73(16):5261-5267. doi:10.1128/AEM.00062-07

14. Weber KA, Achenbach LA, Coates JD. Microorganisms pumping iron: anaerobic microbial iron oxidation and reduction. *Nat Rev Microbiol*. 2006;4(10):752-764. doi:10.1038/nrmicro1490

15. Castro H F, Williams N H, Ogram A. Phylogeny of sulfate-reducing bacteria (1). *FEMS Microbiol Ecol*. 2000;31(1):1-9.

16. Emerson D, Fleming EJ, McBeth JM. Iron-oxidizing bacteria: An environmental and genomic perspective. *Annu Rev Microbiol*. 2010;64(1):561-583. doi:10.1146/annurev.micro.112408.134208

17. Brenner DJ, Krieg NR, Staley JT, Garrity GM, eds. *Bergey's Manual of Systematic Bacteriology, 2nd Edition, Volume 2, Parts A, B, C*. 2nd Editio. New York, NY: Springer-Verlag; 2005.

18. Cleary A, Newsome L, Shaw S, et al. Bioremediation of strontium and technetium contaminated groundwater using glycerol phosphate. *Chem Geol*. 2019;509:213-222.

19. Parkhurst DL, Appelo CAJ. USER'S GUIDE TO PHREEQC (VERSION 2)— A COMPUTER PROGRAM FOR SPECIATION, BATCH-REACTION, ONE-DIMENSIONAL TRANSPORT, AND INVERSE GEOCHEMICAL CALCULATIONS. 1999;(Version 2).

20. Tobler DJ, Cuthbert MO, Greswell RB, et al. Comparison of rates of ureolysis between Sporosarcina pasteurii and an indigenous groundwater community under conditions required to precipitate large volumes of calcite. *Geochim Cosmochim Acta*. 2011;75(11):3290-3301. doi:10.1016/j.gca.2011.03.023

21. Littlewood JL, Shaw S, Peacock CL, Bots P, Trivedi D, Burke IT. Mechanism of Enhanced Strontium Uptake into Calcite via an Amorphous Calcium Carbonate Crystallization Pathway. *Cryst Growth Des*. 2017;17(3):1214-1223. doi:10.1021/acs.cgd.6b01599
